# Supplementary material for: Cell-Free Nucleic Acids for Early Diagnosis of Acute Ischemic Stroke: A Systematic Review and Meta-Analysis
Source: Int J Mol Sci. 2025 Feb 12;26(4):1530. doi: 10.3390/ijms26041530 (PMC11855205; doi:10.3390/ijms26041530)
Supplement: Supplementary file 1 [file ijms-26-01530-s001.zip › ijms-3455617-supplementary.pdf]

S1. Search strategy

PubMed (1971- May 10, 2023)

1. "Cell-Free Nucleic Acids"[Mesh] OR "cfDNA*"[tiab] OR "cirDNA"[tiab] OR "Cf-mtDNA*"[tiab] OR "CfmtDNA*"[tiab] OR "ccf-mtDNA*"[tiab] OR "cf-nDNA*"[tiab] OR "cf-NA*"[tiab] OR "cfRNA*"[tiab] OR "cirRNA*"[tiab]

2. (Nucleic Acid[MeSH] OR "Nucleic Acid*" OR "DNA" OR "DNAs" OR "Deoxyribonucleic Acid*"[tiab] OR "mtDNA*"[tiab] OR "Epigenomic*"[tiab] OR "methyl*"[tiab] OR "epigen*"[tiab] OR "CpG Island*"[tiab] OR "CpG"[tiab] OR "CGI"[tiab] OR "histone modification*"[tiab] OR "topolog*"[tiab] OR "fragment*"[tiab] OR "eccDNA*"[tiab] OR "ssDNA*"[tiab] OR "dsDNA*"[tiab]

3. 1 OR 2

4. Blood[MeSH] OR blood[tiab] OR plasma[tiab] OR serum[tiab]

5. "stroke"[Mesh] OR "Cerebrovascular Disorders"[Mesh] OR "stroke*"[tiab] OR "Apoplex*"[tiab] OR "brain infarct*"[tiab] OR "cerebral infarct*"[tiab] OR "intracranial infarct*"[tiab] OR "Cerebral Circulation Infarct*"[tiab] OR "Brain Ischemia*"[tiab] OR "cerebral Ischemia*"[tiab] OR "intracranial Ischemia*"[tiab] OR "Brain Ischaemia*"[tiab] OR "cerebral Ischaemia*"[tiab] OR "intracranial Ischaemia*"[tiab] OR"cerebral Thrombosis"[tiab] OR "Cerebral Embolism"[tiab] OR "Brain Thrombosis"[tiab] OR "Brain Embolism"[tiab] OR "intracranial Thrombosis"[tiab] OR "intracranial Embolism"[tiab] OR "brain vascular accident*"[tiab] OR "brain vascular dis*"[tiab] OR "brain vascular Occlusion*"[tiab] OR "brain arterial accident*"[tiab] OR "brain arterial dis*"[tiab] OR "brain arterial Occlusion*"[tiab] OR "cerebrovascular accident*"[tiab] OR "cerebrovascular dis*"[tiab] OR "Cerebrovascular Occlusion*"[tiab] OR "cerebral vascular accident*"[tiab] OR "cerebral vascular dis*"[tiab] OR "cerebral vascular Occlusion*"[tiab] OR "cerebral arterial accident*"[tiab] OR "cerebral arterial dis*"[tiab] OR "cerebral arterial Occlusion*"[tiab] OR "intracranial arterial accident*"[tiab] OR "intracranial arterial dis*"[tiab] OR "intracranial arterial Occlusion*"[tiab] OR "intracranial vascular accident*"[tiab] OR "intracranial vascular dis*"[tiab] OR "intracranial vascular Occlusion*"[tiab] OR "CVA"[tiab] OR "CVAs"[tiab]

6. (Biomarkers[MeSH] OR biomarker*[tiab] OR "biological marker*"[tiab] OR "biologic marker*"[tiab] OR "serum marker*"[tiab] OR "biochemical marker*"[tiab] OR "laboratory marker*"[tiab]) AND (Diagnosis[MeSH] OR diagnos*[tiab] OR differentiat*[tiab])

7. (Biomarkers[MeSH] OR biomarker*[tiab] OR "biological marker*"[tiab] OR "biologic marker*"[tiab] OR "serum marker*"[tiab] OR "biochemical marker*"[tiab] OR "laboratory marker*"[tiab]) AND (Prognosis[MeSH] OR prognos*[tiab] OR predict*[tiab] OR Incidence*[tiab] OR mortality*[tiab] OR follow up stud*[tiab] OR course*[tiab] OR rankin*[tiab] OR outcome*[tiab] OR NIHSS[tiab] OR recurrence[tiab])
8. 6 OR 7

9. 3 AND 4 AND 5 AND 8

Web of Science (1900- May 10, 2023)

1. TS=("Cell-Free Nucleic Acids" OR "cfDNA*" OR "cirDNA*" OR "Cf-mtDNA*" OR "CfmtDNA*" OR "ccf-mtDNA*" OR "cf-nDNA*" OR "cf-NA*" OR "cfRNA*" OR "cirRNA*")

2. TS=("Circul*" OR "cell-free" OR "cell free" OR "extracell*" OR "plasma" OR "serum" OR "Extracellular Vesicles" OR "exosom*" OR "vesicle*" OR "vesicular*" OR "Microparticles" OR "EV" OR "EVs" OR "Apoptotic*" OR "Microvesicle*" OR "ectosome*" OR "Exovesicle*")

3. TS=("Nucleic Acid$" OR "DNA$" OR "Deoxyribonucleic Acid*" OR "mtDNA$" OR "Epigenomic*" OR "methyl*" OR "epigen*" OR "CpG Island$" OR "CpG" OR "CGI" OR "histone modification$" OR "topolog*" OR "fragment$" OR "eccDNA$" OR "ssDNA$" OR "dsDNA$" OR "motif*" OR "End sequence$" OR "End site$" OR "Protruding" OR "jagged$" OR "Blunt$" OR "Nucleosom$" OR "RNA$" OR "ribonucleic acid$" OR "circRNA$" OR "ciRNA$" OR "ncRNA$" OR "LncRNA$" OR "SncRNA$" OR "miR*" OR "miRNA$" OR "MicroRNA$" OR "Micro-RNA$" OR "pri-miRNA$" OR "piRNA$" OR "YRNA$" OR "vtRNA$" OR "siRNA$" OR "mRNA$" OR "snRNA$" OR "snoRNA$" OR "npcRNA$" OR "LincRNA$" OR "stRNA$" OR "nmRNA$" OR "shRNA$" OR "scnRNA$" OR "tasiRNA$" OR "scaRNA$" OR "exRNA$")

4. TS=(blood OR plasma OR serum)

5. TS=("stroke$" OR "Apoplex*" OR "brain infarct$" OR "cerebral infarct$" OR "intracranial infarct$" OR "Cerebral Circulation Infarct$" OR "Brain Isch$emia*" OR "cerebral Isch$emia*" OR "intracranial Isch$emia*" OR "cerebral Thrombosis" OR "Cerebral Embolism" OR "Brain Thrombosis" OR "Brain Embolism" OR "intracranial Thrombosis" OR "intracranial Embolism" OR "brain vascular accident$" OR "brain vascular dis*" OR "brain vascular Occlusion$" OR "brain arterial accident$" OR "brain arterial dis*" OR "brain arterial Occlusion$" OR "cerebrovascular accident$" OR "cerebrovascular dis*" OR "Cerebrovascular Occlusion$" OR "cerebral vascular accident$" OR "cerebral vascular dis*" OR "cerebral vascular Occlusion$" OR "cerebral arterial accident$" OR "cerebral arterial dis*" OR "cerebral arterial Occlusion$" OR "intracranial arterial accident$" OR "intracranial arterial dis*" OR "intracranial arterial Occlusion$" OR "intracranial vascular accident$" OR "intracranial vascular dis*" OR "intracranial vascular Occlusion$" OR "CVA$")

6. TS=((“biomarker$” OR "biological marker$" OR "biologic marker$" OR "serum marker$" OR "plasma marker$" OR "blood marker$" OR "biochemical marker$" OR "laboratory marker$") AND (“predict*” OR“diagnos*” OR “differentiat*” OR

“prognos*” OR “predict*” OR “Incidence*” OR “mortality*” OR “follow up stud*” OR “course*” OR “rankin*” OR “outcome*” OR “NIHSS” OR “recurrence”))

7. 2 AND 3

8. 1 OR 7

9. 4 AND 5 AND 6 AND 8

EMBASE (via OVID) (1974- May 10, 2023)

1. exp 'Cell-Free Nucleic Acids'/ or 'Cell-Free Nucleic Acids'.ab,ti. or 'cfDNA*'.ab,ti. or 'cirDNA'.ab,ti. or 'Cf-mtDNA*'.ab,ti. or 'CfmtDNA*'.ab,ti. or 'ccf-mtDNA*'.ab,ti. or 'cf-nDNA*'.ab,ti. or 'cf-NA*'.ab,ti. or 'cfRNA*'.ab,ti. or 'cirRNA*'.ab,ti.

2. exp 'Nucleic Acid'/ or 'Nucleic Acid*'.mp. or 'DNA'.mp. or 'DNAs'.mp. or 'Deoxyribonucleic Acid*'.ab,ti. or 'mtDNA*'.ab,ti. or 'Epigenomic*'.ab,ti. or 'methyl*'.ab,ti. or 'epigen*'.ab,ti. or 'CpG Island$'.ab,ti. or 'CpG'.ab,ti. or 'CGI'.ab,ti. or 'histone modification*'.ab,ti. or 'topolog*'.ab,ti. or 'fragment*'.ab,ti. or 'eccDNA*'.ab,ti. or 'ssDNA*'.ab,ti. or 'dsDNA*'.ab,ti. or 'motif*'.ab,ti. or 'End

sequence*'.ab,ti. or 'End site*'.ab,ti. or 'Protruding'.ab,ti. or 'jagged*'.ab,ti. or 'Blunt*'.ab,ti. or 'Nucleosom*'.ab,ti. or 'RNA'.mp. or 'RNAs'.mp. or 'ribonucleic acid*'.mp. or 'circRNA*'.ab,ti. or 'ciRNA*'.ab,ti. or 'ncRNA*'.ab,ti. or 'LncRNA*'.ab,ti. or 'SncRNA*'.ab,ti. or 'miR'.ab,ti. or 'miRNA*'.ab,ti. or 'MicroRNA*'.ab,ti. or 'Micro-RNA*'.ab,ti. or 'pri-miRNA*'.ab,ti. or 'piRNA*'.ab,ti. or 'YRNA*'.ab,ti. or 'vtRNA*'.ab,ti. or 'siRNA*'.ab,ti. or 'mRNA*'.ab,ti. or 'snRNA*'.ab,ti. or 'snoRNA*'.ab,ti. or 'npcRNA*'.ab,ti. or 'LincRNA*'.ab,ti. or 'stRNA*'.ab,ti. or 'nmRNA*'.ab,ti. or 'shRNA*'.ab,ti. or 'scnRNA*'.ab,ti. or 'tasiRNA*'.ab,ti. or 'scaRNA*'.ab,ti. or 'exRNA*'.ab,ti. [mp=title, abstract, heading word, drug trade name, original title, device manufacturer, drug manufacturer, device trade name, keyword heading word, floating subheading word, candidate term word]

3. ('Circul*' or 'cell-free' or 'cell free').mp. or 'extracell*'.ab,ti. or 'plasma'.ab,ti. or 'serum'.ab,ti. or exp 'Extracellular Vesicles'/ or 'exosom*'.ab,ti. or 'vesicle*'.ab,ti. or 'vesicular*'.ab,ti. or 'Microparticles'.ab,ti. or 'EV'.ab,ti. or 'EVs'.ab,ti. or 'Apoptotic*'.ab,ti. or 'Microvesicle*'.ab,ti. or 'ectosome*'.ab,ti. or 'Exovesicle*'.ab,ti. [mp=title, abstract, heading word, drug trade name, original title, device manufacturer, drug manufacturer, device trade name, keyword heading word, floating subheading word, candidate term word]

4. 2 and 3

5. 1 and 4

6. exp Blood/ or blood.ab,ti. or plasma.ab,ti. or serum.ab,ti.

7. exp Biomarkers/ or biomarker*.ab,ti. or 'biological marker*'.ab,ti. or 'biologic marker*'.ab,ti. or 'serum marker*'.ab,ti. or 'biochemical marker*'.ab,ti. or 'laboratory marker*'.ab,ti.

8. exp Diagnosis/ or diagnos*.ab,ti. or differentiat*.ab,ti. or exp Prognosis/ or prognos*.ab,ti. or predict*.ab,ti. or Incidence*.ab,ti. or mortality*.ab,ti. or follow up stud*.ab,ti. or course*.ab,ti. or rankin*.ab,ti. or outcome*.ab,ti. or NIHSS.ab,ti. or recurrence.ab,ti.

9. exp 'stroke'/ or exp 'Cerebrovascular Disorders'/ or 'stroke*'.ab,ti. or 'Apoplex*'.ab,ti. or 'brain infarct*'.ab,ti. or 'cerebral infarct*'.ab,ti. or 'intracranial infarct*'.ab,ti. or 'Cerebral Circulation Infarct*'.ab,ti. or 'Brain Ischemia*'.ab,ti. or 'cerebral Ischemia*'.ab,ti. or 'intracranial Ischemia*'.ab,ti. or 'Brain Ischaemia*'.ab,ti. or 'cerebral Ischaemia*'.ab,ti. or 'intracranial Ischaemia*'.ab,ti. or 'cerebral Thrombosis'.ab,ti. or 'Cerebral Embolism'.ab,ti. or 'Brain Thrombosis'.ab,ti. or 'Brain Embolism'.ab,ti. or 'intracranial Thrombosis'.ab,ti. or 'intracranial Embolism'.ab,ti. or 'brain vascular accident*'.ab,ti. or 'brain vascular dis*'.ab,ti. or 'brain vascular Occlusion*'.ab,ti. or 'brain arterial accident*'.ab,ti. or 'brain arterial dis*'.ab,ti. or 'brain arterial Occlusion*'.ab,ti. or 'cerebrovascular accident*'.ab,ti. or 'cerebrovascular dis*'.ab,ti. or 'Cerebrovascular Occlusion*'.ab,ti. or 'cerebral vascular accident*'.ab,ti. or 'cerebral vascular dis*'.ab,ti. or 'cerebral vascular Occlusion*'.ab,ti. or 'cerebral arterial accident*'.ab,ti. or 'cerebral arterial dis*'.ab,ti. or 'cerebral arterial Occlusion*'.ab,ti. or 'intracranial arterial accident*'.ab,ti. or 'intracranial arterial dis*'.ab,ti. or 'intracranial arterial Occlusion*'.ab,ti. or 'intracranial vascular accident*'.ab,ti. or 'intracranial vascular dis*'.ab,ti. or 'intracranial vascular Occlusion*'.ab,ti. or 'CVA'.ab,ti. or 'CVAs'.ab,ti.

10. 7 and 8

11. 5 and 6 and 9 and 10

Cochrane Library (1973- May 10, 2023)

1. [mh "Cell-Free Nucleic Acids"] OR (Cell-Free Nucleic Acids):ti,ab,kw OR (cfDNA*):ti,ab,kw OR (cirDNA):ti,ab,kw OR (Cf-mtDNA*):ti,ab,kw OR (CfmtDNA*):ti,ab,kw OR (ccf-mtDNA*):ti,ab,kw OR (cf-nDNA*):ti,ab,kw OR (cf-NA*):ti,ab,kw OR (cfRNA*):ti,ab,kw OR (cirRNA*):ti,ab,kw

2. [mh "Nucleic Acid"] OR (Nucleic Acid*) OR (DNA) OR (DNAs) OR (Deoxyribonucleic Acid*):ti,ab,kw OR (mtDNA*):ti,ab,kw OR (Epigenomic*):ti,ab,kw OR (methyl*):ti,ab,kw OR (epigen*):ti,ab,kw OR (CpG Island$):ti,ab,kw OR (CpG):ti,ab,kw OR (CGI):ti,ab,kw OR (histone modification*):ti,ab,kw OR (topolog*):ti,ab,kw OR (fragment*):ti,ab,kw OR (eccDNA*):ti,ab,kw OR (ssDNA*):ti,ab,kw OR (dsDNA*):ti,ab,kw OR (motif*):ti,ab,kw OR (End sequence*):ti,ab,kw OR (End site*):ti,ab,kw OR (Protruding):ti,ab,kw OR (jagged*):ti,ab,kw OR (Blunt*):ti,ab,kw OR (Nucleosom*):ti,ab,kw OR (RNA' OR 'RNAs' OR 'ribonucleic acid*' OR 'circRNA*):ti,ab,kw OR (ciRNA*):ti,ab,kw OR (ncRNA*):ti,ab,kw OR (LncRNA*):ti,ab,kw OR (SncRNA*):ti,ab,kw OR (miR):ti,ab,kw OR (miRNA*):ti,ab,kw OR (MicroRNA*):ti,ab,kw OR (Micro-RNA*):ti,ab,kw OR (pri-miRNA*):ti,ab,kw OR (piRNA*):ti,ab,kw OR (YRNA*):ti,ab,kw OR (vtRNA*):ti,ab,kw OR (siRNA*):ti,ab,kw OR (mRNA*):ti,ab,kw OR (snRNA*):ti,ab,kw OR (snoRNA*):ti,ab,kw OR (npcRNA*):ti,ab,kw OR (LincRNA*):ti,ab,kw OR (stRNA*):ti,ab,kw OR (nmRNA*):ti,ab,kw OR (shRNA*):ti,ab,kw OR (scnRNA*):ti,ab,kw OR (tasiRNA*):ti,ab,kw OR (scaRNA*):ti,ab,kw OR (exRNA*):ti,ab,kw

3. (Circul*) OR (cell-free) OR (cell free) OR (extracell*):ti,ab,kw OR (plasma):ti,ab,kw OR (serum):ti,ab,kw OR [mh "Extracellular Vesicles"] OR (exosom*):ti,ab,kw OR (vesicle*):ti,ab,kw OR (vesicular*):ti,ab,kw OR (Microparticles):ti,ab,kw OR (EV):ti,ab,kw OR (EVs):ti,ab,kw OR (Apoptotic*):ti,ab,kw OR (Microvesicle*):ti,ab,kw OR (ectosome*):ti,ab,kw OR (Exovesicle*):ti,ab,kw

4. 2 AND 3

5. 1 OR 4

6. [mh "Blood"] OR (blood):ti,ab,kw OR (plasma):ti,ab,kw OR (serum):ti,ab,kw

7. [mh "stroke"] OR [mh "Cerebrovascular Disorders"] OR (stroke*):ti,ab,kw OR (Apoplex*):ti,ab,kw OR (brain infarct*):ti,ab,kw OR (cerebral infarct*):ti,ab,kw OR (intracranial infarct*):ti,ab,kw OR (Cerebral Circulation Infarct*):ti,ab,kw OR (Brain Ischemia*):ti,ab,kw OR (cerebral Ischemia*):ti,ab,kw OR (intracranial Ischemia*):ti,ab,kw OR (Brain Ischaemia*):ti,ab,kw OR (cerebral Ischaemia*):ti,ab,kw OR (intracranial Ischaemia*):ti,ab,kw OR (cerebral Thrombosis):ti,ab,kw OR(Cerebral Embolism):ti,ab,kw OR (Brain Thrombosis):ti,ab,kw OR (Brain Embolism):ti,ab,kw OR (intracranial Thrombosis):ti,ab,kw OR (intracranial Embolism):ti,ab,kw OR (brain vascular accident*):ti,ab,kw OR (brain vascular dis*):ti,ab,kw OR (brain vascular Occlusion*):ti,ab,kw OR (brain arterial accident*):ti,ab,kw OR (brain arterial dis*):ti,ab,kw OR (brain arterial Occlusion*):ti,ab,kw OR (cerebrovascular accident*):ti,ab,kw OR (cerebrovascular dis*):ti,ab,kw OR (Cerebrovascular Occlusion*):ti,ab,kw OR (cerebral vascular accident*):ti,ab,kw OR (cerebral vascular dis*):ti,ab,kw OR (cerebral vascular Occlusion*):ti,ab,kw OR (cerebral arterial accident*):ti,ab,kw OR (cerebral arterial dis*):ti,ab,kw OR (cerebral arterial Occlusion*):ti,ab,kw OR (intracranial arterial accident*):ti,ab,kw OR (intracranial arterial dis*):ti,ab,kw OR (intracranial arterial Occlusion*):ti,ab,kw OR (intracranial vascular accident*):ti,ab,kw OR (intracranial vascular dis*):ti,ab,kw OR (intracranial vascular Occlusion*):ti,ab,kw OR (CVA):ti,ab,kw OR (CVAs):ti,ab,kw

8. [mh "Biomarkers"] OR (biomarker*):ti,ab,kw OR (biological marker*):ti,ab,kw OR (biologic marker*):ti,ab,kw OR (serum marker*):ti,ab,kw OR (biochemical marker*):ti,ab,kw OR (laboratory marker*):ti,ab,kw

9. [mh "Diagnosis"] OR (diagnos*):ti,ab,kw OR (differentiat*):ti,ab,kw OR [mh "Prognosis"] OR (prognos*):ti,ab,kw OR (predict*):ti,ab,kw OR (Incidence*):ti,ab,kw OR (mortality*):ti,ab,kw OR (follow up stud*):ti,ab,kw OR (course*):ti,ab,kw OR (rankin*):ti,ab,kw OR (outcome*):ti,ab,kw OR (NIHSS):ti,ab,kw OR (recurrence):ti,ab,kw

10. 8 AND 9

11. 5 AND 6 AND 7 AND 10

S2. Risk of Bias and Application Concerns in QUADAS-2
Risk of bias--Patient Selection (Could the selection of patients have introduced bias?)

Describe methods of patient selection.

1. Was a consecutive or random sample of patients enrolled? Yes/No/Unclear

2. Did the study avoid inappropriate exclusions (exclusion of hypertension, hyperlipoidemia, diabetes, atrial fibrillation and smoking)? Yes/No/Unclear

Risk of bias--Index Test (Could the conduct or interpretation of the index test have introduced bias?)

Describe the biomarker test and how it was conducted and interpreted.

1.Were the biomarker test results interpreted without knowledge of the results of the reference standard? Yes/No/Unclear

Risk of bias--Reference Standard (Could the reference standard, its conduct, or its interpretation have introduced bias?)

Describe the reference standard and how it was conducted and interpreted.

1.Is the reference standard likely to correctly classify stroke basing on history and physical examination? Yes/No/Unclear

2.Were the reference standard results interpreted without knowledge of the results of biomarker test? Yes/No/Unclear

Risk of bias--Flow and Timing (Could the patient flow have introduced bias?)

Describe any patients who did not receive the biomarker test and/or reference standard.

1.Did all patients (not include healthy control) receive a reference standard? Yes/No/Unclear

2.Did patients (not include healthy control) receive the same reference standard? Yes/No/Unclear

3.Were all patients (not include healthy control) included in the analysis? Yes/No/Unclear

Applicability concern--Patient Selection

Describe included patients (prior testing, presentation and setting).

(Prior testing is disease history taking and physical examination. Presentation is stroke-like symptoms. Setting is ED or neurology department or internal medicine ward.)

1. Is there concern that the included patients do not match the review question in the aspect of prior testing, presentation and setting? Low/High/Unclear

Applicability concern--Index Test

1. Is there concern that biomarker test, its conduct, or interpretation differ from the review question? Low/High/Unclear

Applicability concern--Reference Standard

1. Is there concern that stroke as defined by the reference standard does not match the review question? Low/High/Unclear

S3. Funnel plot analysis of blood biomarkers differentiating ischemic stroke from controls within 24 hours of symptom onset

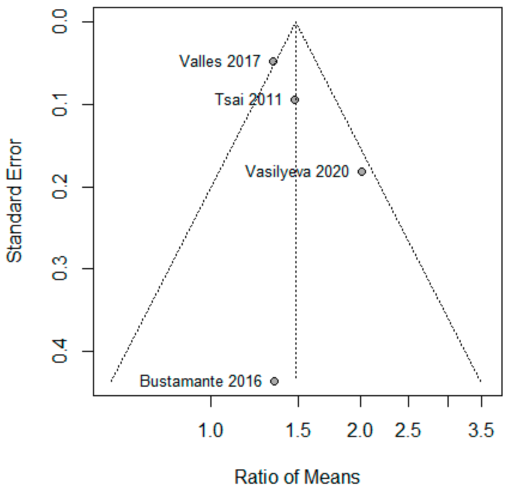

Supplemental Table S1. Basic information of included studies

Study ID	Country	Race	Study design	Sample size	Groups	Cohorts			Age	Sex	Hypertension (%)	DM (%)	Hyperlipidemia (%)	Current smokers (%)
						Discovery	Validation	Replication	(year)	(Male %)				
AdlySadik 2021[1]	Egypt	--	Case Con	96	IS (46) vs Con (50)	Yes	No	No	IS: 58.3 ± 2.1<br>HC: 56.8 ±3.1<br>p = 0.080	IS: 54.3<br>Con: --	IS: 41.3<br>Con: 0	IS: 19.6<br>Con: 0	--	IS: 13.0<br>Con: 0
Chen 2017[2]	China	Mongoloid/Asian	Case Con	83	IS (50) vs Con (33)	Yes	No	No	IS: 64.0 ± 9.4<br>Con: 63.0 ± 7.9<br>p = 0.670	IS: 64.0<br>Con: 61.0<br>p = 0.750	IS: 76.0<br>Con: 75.8<br>p = 0.980	IS: 36.0<br>Con: 9.1<br>p <0.05	IS: 36.0<br>Con: 9.1<br>p = 0.980	--
Chen 2018[3]	China	--	Case Con	230	IS (128) vs Con (102)	Yes	No	No	IS: 68.4 ± 17.3<br>Con: 65.4 ± 16.3	IS: 85.2<br>Con: 72.5	IS: 88.3<br>Con: 66.7	IS: 42.9<br>Con: 37.3	IS: 76.6<br>Con: 33.3	IS: 49.2<br>Con: 49.2
Cheng 2018[4]	China	--	Case Con	119	IS (77) vs Con (42)	Yes	No	No	IS: 61.0 ± 10.4<br>Con: 59.0 ± 4.7<br>p > 0.050	IS: 70.0<br>Con: 69.0<br>p = 1.000	IS: 70.1<br>Con: 47.6<br>p = 0.015	IS: 19.5<br>Con: 14.3<br>p = 0.478	--	IS: 37.7<br>Con: 35.7<br>p = 0.833
Ewida 2021[5]	Egypt	--	Case Con	110	IS (trombotic, 25) vs IS (embolic, 25) vs HS (25) vs HC (35)	Yes	No	No	IS (trombotic): 57.0 (47.0)<br>IS (embolic): 64.0 (40.0)<br>HS: 64.0 (50.0)<br>HC: 57.0 (29.0)<br>p = 0.421	IS (trombotic): 76.0<br>IS (embolic): 64.0<br>HS: 64.0<br>HC: 68.0<br>p = 0.776	IS (trombotic): 52.0<br>IS (embolic): 60.0<br>HS: 64.0<br>HC: 0.0<br>p < 0.001	IS (trombotic): 32.0<br>IS (embolic): 32.0<br>HS: 52.0<br>HC: 0.0<br>p = 0.001	--	IS (trombotic): 64.0<br>IS (embolic): 20.0<br>HS: 20.0<br>HC: 32.0<br>p = 0.002
Eyileten 2022[6]	Poland	--	Case Con	63	IS (28) vs Con (35)	Yes	No	No	IS: 66.4 ± 15.9<br>Con: 65.1 ± 8.0	IS: 46.4<br>Con: 40.0	IS: 64.0<br>Con: 63.0	IS: 17.0<br>Con: 20.0	--	IS: 39.0<br>Con: 39.0

Feng 2019[7]	China	--	Case Con	251	IS (126) vs RC (125)	Yes	No	No	IS: 61.9 ± 10.4<br>RC: 61.0 ± 8.9<br>p = 0.881	IS: 70.6<br>RC: 69.6<br>p = 0.858	IS: 91.3<br>RC: 86.4%<br>p = 0.220	IS: 19.8<br>RC: 15.2<br>p = 0.334	IS: 43.7<br>RC: 39.2<br>p = 0.474	IS: 50.0<br>RC: 51.2<br>p = 0.849
Ishida 2020[8]	Japan	Mongoloid/Asian	Case Con	97	IS (GO, 31) vs IS (PO, 44) vs RC (22)	Yes	No	No	IS (GO): 70.0±0.9<br>IS (PO): 73.5±1.7<br>p = 0.190<br>RC 60.8±4.09	IS (GO): 54.9<br>IS (PO): 41.0<br>RC: 59<br>p = 0.340	IS (GO): 57.0<br>IS (PO): 57.0<br>p = 0.980	IS (GO): 25.8<br>IS (PO): 27.3<br>p = 0.950	--	IS (GO): 29.0<br>IS (PO): 39.0<br>p = 0.880
Ji 2016[9]	China	--	Case Con	131	IS (65) vs Con (66)	Yes	No	No	IS: 64 (16)<br>Con: 60 (9)<br>p = 0.130	IS: 61.5<br>Con: 54.5<br>p = 0.420	IS: 66.2<br>Con: 53.0<br>p = 0.130	IS: 27.7<br>Con: 18.2<br>p = 0.200	IS: 27.7<br>Con: 24.2<br>p = 0.660	--
Jia 2015[10]	China	--	Case Con	242	IS (146) vs Con (96)	Yes	No	No	IS: 67.3 ± 14.2<br>Con: 63.2 ± 15.2	IS: 76.7<br>Con: 66.7	IS: 86.3<br>Con: 72.9	IS: 39.1<br>Con: 34.3	IS: 74.6<br>Con: 35.4	IS: 44.5<br>Con: 43.7
Jin 2017[11]	China	Mongoloid/Asian	Prospective	236	IS (116) vs RC (120)	Yes: IS (10) vs RC (10)	Yes: IS (106) vs RC (110)	No	Discovery<br>IS: 59.8 ± 8.9<br>RC: 61.1 ± 9.4<br>p = 0.755<br>Validation<br>IS: 60.8 ± 9.7<br>RC: 58.6 ± 15.2<br>p = 0.205	Discovery:<br>IS: 50.0<br>RC: 50.0<br>p = 1.000<br>Validation<br>IS: 45.0<br>RC: 54.0<br>p = 0.220	Discovery:<br>IS: 80.0<br>RC: 70.0<br>p = 0.606<br>Validation:<br>IS: 76.0<br>RC: 77.0<br>p = 0.881	Discovery:<br>IS: 20.0<br>RC: 20.0<br>p = 1.000<br>Validation:<br>IS: 32.0<br>RC: 24.0<br>p = 0.881	Discovery:<br>IS: 60.0<br>RC: 70.0<br>p = 0.639<br>Validation:<br>IS: 49.0<br>RC: 55.0<br>p = 0.347	Discovery:<br>IS: 30.0<br>RC: 30.0<br>p = 1.000<br>Validation:<br>IS: 25.0<br>RC: 35.0<br>p = 0.107
Kijpaisalratana 2020[12]	Thailand	Mongoloid/Asian	Case Con	58	IS (PCI, 23) vs SM (35)	Yes	No	No	IS (PCI): 64.5 ±11.8<br>SM: 63.7 ±9.4<br>p = 0.766	IS (PCI): 78.3<br>SM: 17.1<br>p = 0.001	IS (PCI): 65.2<br>SM: 51.4<br>p = 0.300	IS (PCI): 52.2<br>SM: 17.1<br>p = 0.005	--	IS (PCI): 26.1<br>SM: 0.0<br>p = 0.001

Leung 2014[13]	Hong Kong	--	Case Con	116	Stroke (93): IS (74), HS (19) vs HC (23)	Yes	No	No	Stroke: 72 (18) HC: 65	Stroke: 50.5 HC: 69.6	Stroke: 74.2 HC: 0.0	Stroke: 34.4 HC: 0.0	Stroke: 18.3 HC: 0.0	Stroke: 15.1 HC: 0.0
Li 2017[14]	China	Mongoloid/Asian	Case Con	52	IS (Acute, 27) vs Con (25)	Yes	No	No	IS(Acute): 56.5 ± 5.77 Con: 55.0 ± 6.64	IS (Acute): 63.0 Con: 76.0	IS (Acute): 55.6 Con: 28.0	IS (Acute): 18.5 Con: 20.0	--	IS (Acute): 55.6 Con: 40.0
Li 2020[15]	China	--	Prospective	420	IS (210) vs RC (210)	Yes	No	No	IS: 62.2 ± 13.0 RC: 60.5 ± 10.4 p = 0.138	IS: 75.7 RC: 78.6 p = 0.486	IS: 84.3 RC: 78.1 p = 0.105	IS: 23.8 RC: 19.0 p = 0.234	IS: 49.0 RC: 46.2 p = 0.558	IS: 44.3 RC: 43.8 p = 0.922
Li 2015[16]	China	--	Case Con	199	IS (117) vs RC (82)	Yes: IS (40) vs RC (10)	Yes: IS (24) vs RC (22)	Yes: IS (53) vs RC (50)	IS: 68.0 ± 1.5 RC: 67.0 ± 1.2 p: > 0.05	IS: 58.5 RC: 58.0 p > 0.05	--	--	--	--
Liu 2019[17]	China	--	Case Con	65	IS (40) vs Con (25)	Yes	No	No	--	--	--	--	--	--
Liu 2022[18]	China	Mongoloid/Asian/Guangdong	Case Con	77	IS (45) vs RC (32)	Yes	No	No	IS: 66.4 ± 11.9 RC: 62.7 ± 11.8 p = 0.172	IS: 48.9 RC: 40.6 p = 0.473	IS: 57.8 RC: 75.0 p = 0.118	IS: 31.1 RC: 25.0 p = 0.559	IS: 31.1 RC: 50.0 p = 0.094	IS: 35.6 RC: 21.9 p = 0.196
Liu 2015[19]	China	--	Case Con	42	IS (31) vs Con (11)	Yes	No	No	IS: 66.32 (12.56) Con: --	IS: 67.7 Con: --	IS: 83.9 Con: --	IS: 35.5 Con: --	--	--
Long 2013[20]	China	Mongoloid/Asian	Cross sectional	88	IS (38) vs Con (50)	Yes	No	No	IS: 63.0 ± 6.0 HC: 64.0 ± 6.0	IS: 50.0 HC: 48.0	IS: 18.4.0 HC: 10.0	IS: 13.2 HC: 10.0	IS: 15.8 HC: 12.0	IS: 18.4 HC: 20.0
Ma 2019[21]	China	Mongoloid/Asian	Case Con	53	IS (33) vs HC (20)	Yes	No	No	--	--	--	--	--	--

Nguyen 2020[22]	The Netherlands	--	Case Con	26	IS (9) vs ICH (8) vs<br>SM (9)	Yes: IS (9) vs ICH (8) vs<br>SM (9)	Yes: external cohort, IS (20) vs Con (20)	No	IS: 76 ± 8<br>ICH: 71 ± 14<br>SM: 65 ± 16<br>p = 0.25	IS: 67.0<br>ICH: 38.0<br>SM: 67.0<br>p = 0.43	IS: 67.0<br>ICH: 89.0<br>SM: 22.0<br>p = 0.02	IS: 11.0<br>ICH: 50.0<br>SM: (0.0<br>p 0.02	IS: 67.0<br>ICH: 38.0<br>SM: 33.0<br>p = 0.38	--
O'Connell 2017[23]	USA	--	Case Con	63	IS (43) vs<br>SM (20)	Yes	No	No	IS: 72.5 ± 15.5<br>SM: 58.0 ± 17.0<br>p = 0.001	IS: 41.9.0<br>SM: 55.0<br>p = 0.330	IS: 79.1<br>SM: 85.0<br>p = 0.576	IS: 18.6<br>SM: 35.0<br>p = 0.155	IS: 48.8<br>SM: 65.0<br>p = 0.230	IS: 25.6<br>SM: 10.0<br>p = 0.155
Peng 2015[24]	China	Mongoloid/Asian	Case Con	123	IS (72) vs HC (51)	Yes	No	No	IS: 72.4 (9.2)<br>HC: 70.7 (7.5)	IS: 56.9<br>HC: 58.8	IS: 31.9<br>HC: 0.0	IS: 23.6<br>HC: 0.0	IS: 26.4<br>HC: 0.0	IS: 22.2<br>HC: 17.6
Rahmati 2020[25]	Iran	Mongoloid/Asian	Case Con	104	IS (52) vs<br>RC (52)	Yes	No	No	IS: 69.4 ± 11.2<br>RC: 69.4 ± 11.1<br>p = 0.993	IS: 65.4<br>RC: 65.4<br>p = 0.999	--	--	--	--
Rahmati 2021[26]	Iran	--	Case Con	104	IS (52) vs<br>Con (52)	Yes	No	No	--	--	--	--	--	--
Sheikhbahaei 2019 [27]	Iran	--	Cross sectional	20	IS (DM, 15) vs HC (5)	Yes	No	No	IS(DM): 4.4 ± 12.6<br>HC: 68.2 ± 6.0<br>p > 0.05	IS (DM): 33.3<br>HC: 40.0<br>p > 0.05	--	--	--	--
Song 2021[28]	China	Mongoloid/Asian	Case Con	110	IS (80) vs<br>RC (30)	Yes	No	No	IS: 57.0 ± 21.1<br>RC: 56.0 ± 20.9<br>p = 0.763	IS: 66.3<br>RC: 66.7<br>p = 0.658	IS: 60.0<br>RC: 50.0<br>p = 0.562	IS: 52.5<br>RC: 16.7<br>p = 0.032	IS: 60.0<br>RC: 13.3<br>p = 0.016	--
	China		Case Con	70				No	Discovery<br>--	Discovery<br>--	Discovery<br>--	Discovery<br>--	Discovery<br>--	Discovery<br>--

Tian 2016[29]		Mongoloid/Asian			IS (HACI,40) vs<br>RC (30)	Yes: IS (HACI, 7) vs<br>RC (7)	Yes: IS (HACI, 33) vs<br>RC (23)		Validation<br>IS (HACI): 68.0 ± 13.0<br>RC: 63.7 ± 14.3<br>p = 0.474	Validation<br>IS (HACI): 69.7<br>RC: 73.9<br>p = 0.731	Validation<br>IS (HACI): 66.7<br>RC: 73.9<br>p = 0.562	Validation:<br>IS (HACI): 24.2<br>RC: 13.0<br>p = 0.299	Validation<br>IS (HACI): 42.4<br>RC: 65.2<br>p = 0.093	Validation<br>IS (HACI): 27.2<br>RC: 39.1<br>P = 0.35
Tiedt 2017[30]	Germany	Caucasoid/White	Case Con	492	IS (332) vs<br>Con (160)	Yes: IS (20) vs HC (20)	Yes: IS (40) vs HC (40)	Yes: IS (200) vs TIA (72) vs HC (100)	Discovery<br>IS: 74.7 (9.7)<br>HC: 72.7 (10.1)	Discovery<br>IS: 8 40.0<br>HC: 50.0	Discovery<br>IS: 80.0<br>HC: 50.0	Discovery<br>IS: 20.0<br>HC: 0.0	--	--
									Validation<br>IS 74.7 (13.8)<br>HC 69.7 (8.8)	Validation<br>IS: 55.0<br>HC: 40.0	Validation<br>IS: 85.0<br>HC: 65.0	Validation<br>IS: 10.0<br>HC: 5.0	--	--
									Replication<br>IS 74.1 (13.4)<br>HC 65.6 (13.4)<br>TIA 74.8 (12.4)	Replication<br>IS: 56.5<br>HC: 35.0<br>TIA: 45.8	Replication<br>IS: 78.9<br>HC: 35.0<br>TIA: 75.0	Replication<br>IS: 18.2<br>HC: 6.0<br>TIA: 25.4	--	--
Toor 2023[31]	Qatar	--	Case Con	252	IS (191) vs<br>TIA (61)	Yes: IS (96) vs TIA (31)	Yes: IS (95) vs TIA (30)	No	Discovery<br>IS 50.38 ± 9.40<br>TIA 48.23 ± 9.68	Discovery<br>IS: 89.6<br>TIA: 90.3	Discovery<br>IS: 72.2<br>TIA: 38.7	Discovery<br>IS: 49.0<br>TIA: 38.7	--	Discovery<br>IS: 41.7<br>TIA: 54.8
									Validation<br>IS 50.01 ± 9.50<br>TIA 49.43 ± 10.84	Validation<br>IS: 94.7<br>TIA: 86.7	Validation<br>IS: 73.7<br>TIA: 56.7	Validation<br>IS: 47.4<br>TIA: 43.3	--	Validation<br>IS: 46.3<br>TIA: 36.7
Valles 2017[32]	Spain	Caucasoid/White	Case Con	270	IS (243) vs Con (27)	Yes	No	No	IS 70.7 ± 12.2<br>Con: --	IS: 67.4 (Female%)<br>Con: --	IS: 78.0<br>Con: --	IS: 37.0<br>Con: --	IS: 44.7<br>Con: --	IS: 24.5<br>Con: --
Vasilyeva 2020[33]	Russia	Caucasoid/White	Prospective	13	IS (8) vs Con (5)	Yes	No	No	--	--	--	--	--	--

Wang 2017[34]	China	--	Case Con	61	IS (36) vs Con (25)	Yes	No	No	IS: 65.1±10.2<br>HC: 66.4±11.2	IS: 81.0<br>HC: 76.0	--	--	--	--
Wang 2018[35]	China	Mongoloid/Asian	Case Con	72	IS (HIS, 15) vs<br>IS (d1-3, 33) vs Con (24)	Yes	No	No	HIS: 57.7 ± 11.8<br>IS(d1-3): 57.8 ± 10.6<br>Con: 56.6 ± 9.2<br>p > 0.05	HIS: 66.7<br>IS(d1-3): 72.7<br>Con: 75.0<br>p > 0.05	HIS: 46.7<br>IS(d1-3): 66.7*<br>Con: 29.2<br>* p < 0.05 vs Con	HIS: 13.3<br>IS(d1-3): 27.3<br>Con: 20.8<br>p > 0.05	HIS: 26.7<br>IS(d1-3): 45.5<br>Con: 37.5<br>p > 0.05	HIS: 33.3<br>IS(d1-3): 48.5<br>Con: 25.0<br>p > 0.05
Wang 2014[36]	China	--	Prospective	252	IS (136) vs Con (116)	Yes	Yes: same cohort	No	IS: 50 ± 13<br>Con: 53 ± 11	IS (MRI-): 58.3<br>IS (MRI +): 52.6<br>Con: 51.7<br>p > 0.05	--	--	--	--
Wang 2022[37]	China	Mongoloid/Asian	Case Con	438	IS (236) vs<br>TIA (66) vs<br>HC (136)	Yes: IS (36) vs HC (36)	Yes: IS (200) vs TIA (66) vs HC (100)	No	Discovery<br>IS: 61.8 (12.6)<br>HC: 65.8 (11.9)<br>p = 0.177	Discovery<br>IS: 54.6<br>HC: 58.3<br>p = 0.812	Discovery<br>IS: 58.3<br>HC: 44.4<br>p = 0.238	Discovery<br>IS: 25.0<br>HC: 16.7<br>p = 0.384	Discovery<br>IS: 30.6<br>HC: 22.2<br>p = 0.422	--
									Validation<br>IS 64.8 (10.7)<br>TIA: 65.0 (11.0)<br>HC: 64.9 (11.3)<br>p = 0.999	Validation<br>IS: 66.5<br>TIA: 59.1<br>HC: 57.0<br>p = 0.107	Validation<br>IS:67<br>TIA: 60.6<br>HC: 43.0<br>p <0.001	Validation<br>IS: 29.0<br>TIA: 25.8<br>HC: 15.0<br>p = 0.008	Validation<br>IS: 23.5<br>TIA: 36.4<br>HC: 24.0<br>p = 0.923	--
Wang 2017[38]	China	Mongoloid/Asian	Case Con	117	IS (78) vs Con (39)	Yes	No	No	IS: 60.0 ± 10.5<br>Con: 61.0 ± 5.1<br>p = 0.601	IS: 70.5<br>Con: 71.8<br>p = 1.000	IS: 65.4<br>Con: 38.5<br>p = 0.010	IS: 19.2<br>Con: 20.5<br>p = 1.000	IS: 24.4<br>Con: 76.9<br>p <0.001	--

Xiang 2017[39]	China	Mongoloid/Asian	Case Con	85	IS (46) vs Con (39)	Yes: IS (10) vs Con (10)	Yes: IS (46) vs Con (39)	No	IS: 63.1 ± 11.7<br>Con: 65.5 ± 11.4<br>p = 0.150	IS: 63.04<br>Con: 61.54<br>p > 0.05	--	--	--	--
Xiao 2019[40]	China	Mongoloid or Asian	Cross sectional	65	IS (40) vs<br>HC (25)	Yes	No	No	IS: 67.2 ± 9.3<br>HC: 65.7 ± 10.3	IS: 62.5<br>HC: 64.0	--	--	--	--
Xu 2020[41]	China	--	Case Con	200	IS (AMS, 100) vs<br>RC (100)	Yes	Yes	No	IS (AMS): 59 ± 12.7<br>RC: 57 ± 10.1<br>p = 0.590	IS (AMS): 57.0<br>RC: 52.0<br>p = 0.570	IS (AMS): 38.0<br>RC: 43.0<br>p = 0.618	--	IS (AMS): 41<br>RC: 45.0<br>p = 0.668	IS (AMS): 58.0<br>RC: 60.0<br>p = 0.886
Zhang 2020[42]	China	Mongoloid or Asian	Case Con	110	IS (40) vs RC (70)	Yes	No	No	IS: 67.5 ± 11.2<br>RC: 66.2 ± 10.8<br>p =0.461	IS: 61.3<br>RC: 55.7<br>p = 0.522	IS: 67.7<br>RC: 52.9<br>p = 0.071	IS: 24.7<br>RC: 17.1<br>p = 0.253	--	--
Zhang 2020[43]	China	--	Case Con	140	IS (59) vs<br>RC (81)	Yes	No	No	IS: 68.5 ± 10.2<br>RC: 66.7 ± 12.6<br>p = 0.32	IS: 60.6<br>RC: 49.4<br>p = 0.12	IS: 45.5<br>RC: 33.3<br>p = 0.087	IS: 16.7<br>RC: 8.6<br>p = 0.105	--	--
Zhao 2016[44]	China	Mongoloid or Asian	Prospective	272	IS (168) vs<br>HC (104)	Yes	No	No	IS: 70 ±8<br>HC: 69 ±9<br>p = 0.629	IS: 52.3<br>HC: 52.9<br>p = 0.518	IS: 68.5<br>HC: --	IS: 37.5<br>HC: --	IS: 54.2<br>HC: --	IS: 48.8<br>HC: --
Zhao 2019[45]	China	--	Prospective	128	IS (76) vs<br>HC (52)	Yes	No	No	--	--	--	--	--	--
Zhao 2014[46]	China	--	Case Con	110	IS (80) vs<br>Con (30)	Yes	No	No	--	IS: 66.25<br>Con: 66.7<br>p > 0.05	p > 0.05	p > 0.05	p > 0.05	--
Zhou 2018[47]	China	Mongoloid or Asian	Case Con	100	IS (50) vs<br>Con (50)	Yes	No	No	IS: 65.4 ± 10.3<br>Con: 63.3 ± 14.3<br>p > 0.05	IS: 52.0<br>Con: 48.0<br>p > 0.05	IS: 60.0<br>Con: 54.0<br>p > 0.05	IS: 48.0<br>Con: 16.0<br>p < 0.01	IS: 60.0<br>Con: 26.0<br>p < 0.01	--

Zhou 2014[48]	China	--	Case Con	89	IS (68) vs<br>Con (21)	Yes	No	No	IS: 64 ± 3.5<br>Con: 58 ± 2.17<br>p = 0.095	IS: 66.0<br>Con: 48.0	IS: 54.4<br>Con: --	IS: 25.0<br>Con: --	IS: 11.7<br>Con: --	IS: 26.5<br>Con: --
Zhou 2021[49]	China	--	Case Con	216	IS (108) vs Con (108)	Yes	No	No	IS: 66.3 ±11.5<br>Con: 64.5 ±12.8<br>p = 0.260	IS: 51.9<br>Con: 57.4<br>p = 0.410	IS: 36.1<br>Con: 24.1<br>p = 0.050	--	--	IS: 52.8<br>Con: 45.4<br>p = 0.280
Zhou 2022[50]	China	--	Prospective	105	IS (60) vs<br>Con (45)	Yes	No	No	--	--	--	--	--	--
Zuo 2020[51]	China	Mongoloid or Asian	Case Con	378	IS (239) vs<br>Con (139)	Yes: IS (3) vs HC (3)	Yes: IS (36) vs HC (36)	Yes: IS (200) vs HC (100)	Discovery:<br>IS: 63.0 (1.5)<br>Con: 59.3 (2.6)	Discovery:<br>IS: 33.0<br>Con: 33.0	Discovery:<br>IS: 33.0<br>Con: 33.0	Discovery:<br>IS: 0.0<br>Con: 0.0	Discovery:<br>IS: 0.0<br>Con: 0.0	Discovery:<br>IS: 0.0<br>Con: 0.0
									Validation:<br>IS: 66.3 (13.3)<br>Con: 66.4 (13.2)	Validation<br>IS: 53.0<br>Con: 53.0	Validation<br>IS: 72.0<br>Con: 50.0	Validation<br>IS: 47.0<br>Con: 22.0	Validation<br>IS: 17.0<br>Con: 39.0	Validation<br>IS: 25.0<br>Con: 8.3
									Replication:<br>IS: 72 (21)<br>Con: 64 (12)	Replication<br>IS: 67.5<br>Con: 53.0	Replication<br>IS: 76.0<br>Con: 45.0	Replication<br>IS: 32.5<br>Con: 10.0	Replication<br>IS: 26.0<br>Con: 28.0	Replication<br>IS: 25.5<br>Con: 7.0
Rainer 2007[52]	China	Mongoloid or Asian	Prospective	153	IS (118) vs HS (35)	Yes	No	No	IS and HS:<br>74 (15)	IS and HS: 103 (52)	IS and HS: 116 (59)	IS and HS: 46 (23)	IS and HS: 50 (25)	IS and HS: 49 (25)
Bustamante 2016[53]	Spain	--	Prospective	69	IS (nonLAC for IVT, 54): ENI (33), nonENI (21) vs HC (15)	Yes	No	No	IS: 77.0 (12)<br>IS (ENI): 77.5 (10.5)<br>IS (nonENI): 77.0 (26.5)<br>p = 0.804	IS: 44.4<br>IS (ENI): 45.5<br>IS (nonENI): 42.9<br>p = 0.851	IS: 68.5<br>IS (ENI): 22 (66.7%)<br>IS (nonENI): 71.4<br>p = 0.713	IS: 20.4<br>IS (ENI): 24.2<br>IS (nonENI): 14.3<br>p = 0.497	IS: 35.8<br>IS (ENI): 42.4<br>IS (nonENI): 25.0<br>p = 0.200	IS: 13.2<br>IS(ENI): 15.2<br>IS (nonENI): 10.0<br>p = 0.697

Gui 2019[54]	China	--	Case Con	205	IS (CE, 51) vs IS (LAA, 49) vs IS (LAC, 45) vs IS (SUE, 27) vs HC (33)	Yes:<br>IS (CE, 23) vs IS (LAA, 26) vs IS (LAC, 27) vs IS (SUE, 11) vs HC (13)	Yes:<br>IS (CE, 28) vs IS (LAA, 23) vs IS (LAC, 18) vs IS (SUE, 16) vs HC (20)	No	Discovery<br>CE 60 (10)<br>LAA 60 (12)<br>LAC 62 (13)<br>SUE 61 (13)<br>HC 58 (11)	Discovery<br>CE: 43.0<br>LAA: 50.0<br>LAC: 48.0<br>SUE: 45.0<br>HC: 46.0	Discovery<br>CE: 35.0<br>LAA: 54.0<br>LAC: 67.0<br>SUE: 64.0<br>HC: 0.0	Discovery<br>CE: 22.0<br>LAA: 31.0<br>LAC:26.0<br>SUE: 27.0<br>HC: 0.0	Discovery<br>CE: 17.0<br>LAA: 35.0<br>LAC: 18.0<br>SUE: 36.0<br>HC: 0.0	Discovery<br>CE: 13.0<br>LAA: 42.0<br>LAC: 41.0<br>SUE: 36.0<br>HC: 23.0
									Validation<br>CE 57 (14)<br>LAA 63 (16)<br>LAC 65 (11)<br>SUE 60 (8)<br>HC62 (13)	Validation<br>CE: 50.0<br>LAA: 52.0<br>LAC: 50.0<br>SUE: 50.0<br>HC: 50.0	Validation<br>CE: 35.0<br>LAA: 43.0<br>LAC: 67.0<br>SUE: 62.0<br>HC: 0.0	Validation<br>CE: 28.0<br>LAA: 22.0<br>LAC: 33.0<br>SUE: 31.0<br>HC: 0.0	Validation<br>CE: 11.0<br>LAA: 34.0<br>LAC: 17.0<br>SUE: 43.0<br>HC: 0.0	Validation<br>CE: 21.0<br>LAA: 48.0<br>LAC: 55.0<br>SUE: 37.0<br>HC: 25.0
vanKralingen 2019[55]	UK	--	Prospective	173	IS (139) vs SM (34)	Yes: IS (29) vs SM (10)	Yes: IS (139) vs SM (34)	No	Discovery<br>IS: 73.0 (19.0)<br>SM: 70.0 (21.2)<br>p > 0.05	Discovery<br>IS: 75.9<br>SM: 70.0<br>p > 0.05	Discovery<br>IS: 51.7<br>SM: 70.0<br>p > 0.05	Discovery<br>IS: 24.1<br>SM: 30.0<br>p > 0.05	Discovery<br>IS: 31.0<br>SM: 30.0<br>p > 0.05	Discovery<br>IS: 31.0<br>SM: 30.0
									Validation<br>IS: 68.0 (19.0)<br>SM: 63.5 (14.2)<br>p > 0.05	Validation<br>IS: 64.7<br>SM: 55.9<br>p > 0.05	Validation:<br>IS: 41.0<br>SM: 38.2<br>p > 0.05	Validation:<br>IS: 17.3<br>SM: 20.6<br>p > 0.05	Validation:<br>IS: 25.9<br>SM: 17.6<br>p > 0.05	Validation<br>IS: 32.4<br>SM: 26.5

Gong 2016[56]	China	--	Prospective	218	IS (MCI, 88) vs IS (nonMCI, 40) vs ICH (45) vs HC (45)	Yes	No	No	IS (MCI): 58.6 ± 3.2<br>IS (nonMCI): 58.2 ± 2.3<br>ICH: 57.9 ± 2.6<br>HC: 57.8 ± 2.4<br>p = 0.409	IS (MCI): 63.0<br>IS (nonMCI): 65.0<br>ICH: 64.0<br>HC: 58.0<br>p = 0.895	--	--	--	--
Tsai 2011[57]	Taiwan	--	Case Con	100	IS (50) vs Con (50)	Yes	No	No	IS: 67.0 ± 1.3<br>Con: 65.0 ± 1.1<br>p > 0.05	IS: 62.0<br>Con: 58.0<br>p > 0.05	IS: 82.0<br>Con: 76.0<br>p > 0.05	IS: 36.0<br>Con: 32.0<br>p > 0.05	IS: 38.0<br>Con: 64.0<br>p > 0.05	IS: 42.0<br>Con: 30.0<br>p > 0.05
Bai 2018[58]	China	Mongoloid or Asian	Case Con	52	IS (26) vs Con (26)	Yes	No	No	IS: 66.9 ± 2.3<br>Con: 65.4 ± 2.0<br>p = 0.627	IS: 50.0<br>Con: 50.0<br>p = 1.000	IS: 50.0<br>Con: 19.2<br>p = 0.020	IS: 15.4<br>Con: 19.2<br>p = 1.000	--	IS: 15.4<br>Con: 23.1<br>p = 0.482
Wu 2019[59]	China	Mongoloid or Asian	Prospective	142	IS (71) vs RC (71)	Yes	No	No	IS: 67.9 ±1.3<br>RC: 68.6 ±1.1<br>p = 0.676	IS: 83.1<br>RC: 83.1<br>p =1.000	IS: 63.4<br>RC: 49.3<br>p = 0.128	IS: 35.2<br>RC: 19.7<br>p = 0.059	--	IS: 46.5<br>RC: 31.0<br>p = 0.085
Kotb 2019[60]	Egypt	--	Case Con	66	IS (ACI, 44) vs HC (22)	Yes	No	No	IS: 63.69 ± 6.42<br>Con: 64.38 ± 6.23<br>p = 0.501	--	--	--	--	--
Chen 2020[61]	China	--	Prospective	430	IS (215) vs RC (215)	Yes	No	No	IS: 62.7 ± 11.0<br>RC: 61.7 ± 9.3<br>p = 0.325	IS: 72.6<br>RC: 76.7<br>p = 0.318	IS: 86.0<br>RC: 74.9<br>p = 0.004	IS: 29.3<br>RC: 20.9<br>p = 0.045	IS: 49.3<br>RC: 45.1<br>p = 0.385	IS: 47.4<br>RC: 41.9<br>p = 0.244
Yang 2018[62]	China	--	Case Con	141	IS (96) vs NND (45)	Yes	No	No	IS: 61.5 (28.0)<br>NND: --	IS: 62.5<br>NND: --	IS: 54.16<br>NND: --	IS: 27.08<br>NND: --	--	IS: 30.21<br>NND: --

Guo 2022[63]	China	--	Prospective	192	IS (142) vs HC (50)	Yes	No	No	IS: 52.40 ± 2.4<br>HC: 52.2 ± 2.8<br>p = 0.758	IS: 57.7<br>HC: 60.0<br>p = 0.824	IS: 59.9<br>HC: 61.4<br>p = 0.624	IS: 43.0<br>HC: 46.0<br>p = 0.760	IS: 57.7<br>HC: 64.0<br>p = 0.514	IS: 62.7<br>HC: 76.0<br>p = 0.114
Otero-Ortega 2021[64]	Madrid	--	Prospective	72	IS (CSC, 24) vs IS (SC, 24) vs HC (24)	Yes, IS (CSC, 4) vs IS (SC, 4) vs HC (4)	Yes, IS (CSC, 20) vs IS (SC, 20) vs HC (20)	No	--	--	--	--	--	--
Zhou 2022[65]	China	--	Case Con	40	IS (LAA, 12) vs AS (13) vs Con (15)	Yes	No	No	IS (LAA): 64.0 ± 3.4<br>AS: 68.8 ± 3.4<br>Con: 65.5 ± 0.7	IS (LAA): 50.0<br>AS: 31.0<br>Con: 33.0	IS (LAA): 53.8<br>AS: 92.9<br>Con: 0.0	IS (LAA): 50.0<br>AS: 21.4<br>Con: 0.0	--	IS (LAA): 50.0<br>AS: 35.7<br>Con: 26.7
Chen 2021[66]	China	--	Case Con	30	IS (15) vs Con (15)	Yes	No	No	--	--	--	--	--	--

Notes: The normally distributed data were expressed as mean ± SD, while data with a skewed distribution were shown as median (interquartile range). Control (Con), Healthy control (HC), Risk control (RC), Ischemic stroke (IS), Hemorrhagic stroke (HS), Good outcome (GO), Poor outcome (PO), Stroke mimics (SM), Intracerebral hemorrhage (ICH), Hyperacute cerebral infarction (HACI), Hyperacute IS (HIS), Acute minor stroke (AMS), Magnetic Resonance Imaging (MRI), Diabetes Mellitus (DM), Anterior Cerebral Infarction (ACI), Massive cerebral infarction (MCI), Cardioembolism (CE), Posterior cerebral infarction (PCI), Transient ischemic attack (TIA), Intravenous thrombolysis (IVT), Early neurological improvement (ENI), Large-artery atherosclerosis (LAA), Lacunar stroke (LAC), Stroke with undermined etiology (SUE), Non neurological disease (NND), Cortical-Subcortical (CSC), Subcortical (SC), Atherosclerosis (AS).

Supplemental Table S2. QUADAS-2 of included studies

#	No.	Risk of bias				Applicability concern		
		Patient Selection	Index test	Reference Standard	Flow and Timing	Patient Selection	Index Test	Reference Standard
1	AdlySadik 2021[1]	Unclear	Low	Low	Unclear	Low	Unclear	Low
2	Chen 2017[2]	Low	Low	Low	Low	Low	Low	Low
3	Chen 2018[3]	Low	Low	Low	Unclear	Low	Low	Low
4	Cheng 2018[4]	HIgh	Low	Low	Low	Low	Low	Low
5	Ewida 2021[5]	Unclear	Low	Low	Low	Low	Low	Low
6	Eyileten 2022[6]	High	Low	Low	Low	Low	Low	Low
7	Feng 2019[7]	Low	Low	Low	Low	Low	Low	Low
8	Ishida 2020[8]	Low	Low	Low	Unclear	Low	Low	Low
9	Ji 2016[9]	Unclear	Low	Low	Low	Low	Unclear	Low
10	Jia 2015[10]	Unclear	Low	Low	Low	Low	Low	Low
11	Jin 2017[11]	Low	Low	Low	Low	Low	Low	Low
12	Kijpaisalrat ana 2020[12]	Low	Low	Low	Low	Low	Low	Low
13	Leung 2014[13]	Unclear	Low	Low	Low	Low	Low	Low
14	Li 2017[14]	Low	Low	Low	Low	Low	Low	Low
15	Li 2020[15]	Low	Low	Low	Low	Low	Low	Low
16	Li 2015[16]	Unclear	Low	Low	Low	Low	Low	Low
17	Liu 2019[17]	Unclear	Low	Low	Unclear	Low	Low	Low
18	Liu 2022[18]	Unclear	Low	Low	Low	Low	Low	Low
19	Liu 2015[19]	Low	Low	Low	Low	Low	Low	Low
20	Long 2013[20]	High	Low	Low	Low	Low	Low	Low
21	Ma 2019[21]	Unclear	Low	Low	Low	Low	Low	Low

22	Nguyen 2020[22]	High	Low	Low	Low	Low	Low	Low
23	O'Connell 2017[23]	Unclear	Low	Low	Low	Low	Unclear	Low
24	Peng 2015[24]	Unclear	Low	Low	Low	Low	Low	Low
25	Rahmati 2020[25]	Low	Low	Low	Unclear	Low	Unclear	Low
26	Rahmati 2021[26]	Unclear	Low	Low	Unclear	Low	Unclear	Low
27	Sheikhbahaei 2019[27]	Low	Low	Low	Low	Low	Low	Low
28	Song 2021[28]	Low	Low	Low	Low	Low	Low	Low
29	Tian 2016[29]	Low	Low	Low	Low	Low	Unclear	Low
30	Tiedt 2017[30]	Low	Low	Low	Low	Low	Low	Low
31	Toor 2023[31]	Low	Low	Unclear	Unclear	Low	Low	Unclear
32	Valles 2017[32]	Low	Low	Low	Unclear	Low	Low	Low
33	Vasilyeva 2020[33]	Unclear	Low	Low	Unclear	Low	Low	Unclear
34	Wang 2017[34]	Low	Low	Low	Low	Low	Low	Low
35	Wang 2018[35]	Unclear	Low	Low	Low	Low	Low	Low
36	Wang 2014[36]	Low	Low	Low	Low	Unclear	Low	Unclear
37	Wang 2022[37]	Unclear	Low	Low	Low	Low	Low	Low
38	Wang 2017[38]	Low	Low	Low	Unclear	Low	Low	Low
39	Xiang 2017[39]	Unclear	Low	Low	Low	Low	Low	Low
40	Xiao 2019[40]	High	Low	Low	Low	Unclear	Low	Low
41	Xu 2020[41]	Unclear	Low	Low	Low	Low	Low	Low
42	Zhang 2020[42]	Unclear	Low	Low	Low	Low	Low	Low
43	Zhang 2020[43]	Low	Low	Low	Low	Low	Low	Low
44	Zhao 2016[44]	Low	Low	Low	Low	Low	Low	Low
45	Zhao 2019[45]	Unclear	Low	Low	Low	Low	Low	Low

46	Zhao 2014 [46]	Unclear	Low	Low	Unclear	Low	Low	Low
47	Zhou 2018[47]	Unclear	Low	Low	Low	Low	Low	Low
48	Zhou 2014[48]	Unclear	Low	Low	Low	Low	Low	Low
49	Zhou 2021[49]	Unclear	Low	Low	Low	Low	Low	Low
50	Zhou 2022[50]	Unclear	Low	Low	Low	Low	Low	Low
51	Zuo 2020[51]	Unclear	Low	Low	Low	Low	Low	Low
52	Rainer 2007[52]	Low	Low	Low	Low	Low	Low	Low
53	Bustamante 2016[53]	Unclear	Low	Unclear	Unclear	Low	Low	Low
54	Gui 2019[54]	Unclear	Low	Low	Low	Low	Low	Low
55	vanKralingen 2019[55]	Unclear	Low	Low	Low	Low	Low	Low
56	Gong 2016[56]	Unclear	Low	Low	Low	Low	Low	Low
57	Tsai 2011[57]	Unclear	Low	Low	Unclear	Low	Low	Low
58	Bai 2018[58]	Unclear	Low	Low	Low	Low	Unclear	Low
59	Wu 2019[59]	Unclear	Low	Low	Low	Low	Low	Low
60	Kotb 2019[60]	Unclear	Low	Low	Low	Low	Low	Low
61	Chen 2020[61]	Low	Low	Low	Low	Low	Unclear	Low
62	Yang 2018[62]	Unclear	Low	Unclear	Unclear	Low	Low	Low
63	Guo 2022[63]	Unclear	Low	Low	Low	Low	Low	Low
64	Otero-Ortega 2021[64]	Unclear	Low	Low	Low	Low	Low	Low
65	Zhou 2022[65]	High	Low	Low	Low	Low	Low	Low
66	Chen 2021[66]	Unclear	Low	Low	Unclear	Unclear	Low	Low

Supplemental Table S3. The statistical information of cfRNA in the included studies with AUC

NO.	Study ID	Groups	Specimen	Sampling time	Biomarker	Level (fold change)	Association	AUC (95% CI)	Cut point	Sen (%)	Spe (%)	Adjusted OR	Unit	Normalization	Method
1	Peng 2015[24]	IS (68) vs Con (51)	Serum	8 d	let-7e	Up	--	--	--	--	--	--	Relative expression	18S rRNA, $2^{-\Delta\Delta CT}$	RT-qPCR
2	Peng 2015[24]	IS (63) vs Con (51)	Serum	15 d	let-7e	Up	--	--	--	--	--	--	Relative expression	18S rRNA, $2^{-\Delta\Delta CT}$	RT-qPCR
3	Wang 2014[36]	IS (MRI-, 60) vs Con (116)	Plasma	24 h	miR-106b-5p	Up	--	0.999 (0.997-1.000)	--	--	--	--	Relative expression	RNU6B, $2^{-\Delta\Delta CT}$	RT-qPCR
4	Wang 2014[36]	IS (MRI+, 76) vs Con (116)	Plasma	24 h	miR-320d	Down	--	0.987 (0.972-1.000)	--	--	--	--	Relative expression	RNU6B, $2^{-\Delta\Delta CT}$	RT-qPCR
5	Wang 2014[36]	IS (MRI+, 76) vs Con (116)	Plasma	24 h	miR-320e	Down	--	0.981 (0.963-0.998)	--	--	--	--	Relative expression	RNU6B, $2^{-\Delta\Delta CT}$	RT-qPCR
6	Tian 2016[29]	IS (TACI, 4) vs RC (23)	Plasma	6 h	miR-16	Up	--	0.978 (0.925-1.031)	3.093	100	91.3	--	Relative expression	Spiked-in cel-miR-54, $2^{-\Delta CT}$	RT-qPCR
7	Wang 2014[36]	IS (MRI-, 60) vs Con (116)	Plasma	24 h	miR-320d	Down	--	0.977 (0.952-1.000)	--	--	--	--	Relative expression	RNU6B, $2^{-\Delta\Delta CT}$	RT-qPCR
8	Wang 2014[36]	IS (MRI-, 60) vs Con (116)	Plasma	24 h	miR-320e	Down	--	0.953 (0.913-0.994)	--	--	--	--	Relative expression	RNU6B, $2^{-\Delta\Delta CT}$	RT-qPCR
9	Wang 2014[36]	IS (MRI+, 76) vs Con (116)	Plasma	24 h	miR-106b-5p	Up	--	0.962 (0.930-0.993)	--	--	--	--	Relative expression	RNU6B, $2^{-\Delta\Delta CT}$	RT-qPCR

10	Zhou 2021[49]	IS (108) vs Con (108)	Serum	24 h	miR-124	Down	Low miR-124 expression correlated with poor survival prognosis	0.953	--	93.5	91.7	--	Relative expression	U6, $2^{-\Delta\Delta CT}$	RT-qPCR
11	Tian 2016[29]	IS (LAA, 9) vs RC (23)	Plasma	6 h	miR-16	Up	--	0.952 (0.879-1.024)	2.804	100	91.3	--	Relative expression	Spiked-in cel-miR-54, $2^{-\Delta CT}$	RT-qPCR
12	Wang 2014[36]	IS (MRI+, 76) vs Con (116)	Plasma	24 h	miR-4306	Up	--	0.952 (0.922-0.982)	--	--	--	--	Relative expression	RNU6B, $2^{-\Delta\Delta CT}$	RT-qPCR
13	Zhou 2021[49]	IS (108) vs Con (108)	Serum	48 h	miR-124	Down	Low miR-124 expression correlated with poor survival prognosis	0.949		93.5	91.7	--	Relative expression	U6, $2^{-\Delta\Delta CT}$	RT-qPCR
14	AdlySadik 2021[1]	IS (46) vs Con (50)	Serum	24 h	miR-155	Up (8.5)	--	0.940	1.750	85.7	100	--	Relative expression	U6, ΔCT	RT-qPCR
15	Liu 2022[18]	IS (45) vs RC (32)	Plasma	24 h	CircOGDH	Up (54)	Penumra size, r = 0.962, p = 0.002	0.933	--	82.2	96.9	--	Relative expression	GAPDH or β-actin, $2^{-\Delta\Delta CT}$	RT-qPCR
16	Long 2013[20]	IS (38) vs Con (50)	Plasma	24 h	let-7b score	Up (1.713)	--	0.930 (0.879-0.980)	1.675	92.0	84.0	--	Relative expression	U6, $2^{-\Delta\Delta CT}$	RT-qPCR
17	Gui 2019[54]	IS (CE, 51) vs Con (33)	Plasma	24 h	let-7e	Up	--	0.923 (0.859-0.998)	--	89.0	90.0	--	Relative expression	Spiked-in cel-miR-39, $2^{-\Delta\Delta CT}$	RT-qPCR
18	Long 2013[20]	IS (38) vs Con (50)	Plasma	24 h	miR-126 score	Down (1.910)	--	0.920 (0.871-0.978)	1.750	92.0	84.0	--	Relative expression	U6, $2^{-\Delta\Delta CT}$	RT-qPCR

19	Ewida 2021[5]	IS (50) vs HS (25)	Serum	24 h	lncRNA LINK-A	Down	--	0.914	0.280	92.0	94.0	--	Relative expression	GAPDH, $2^{-\Delta\Delta CT}$	RT-qPCR
20	Long 2013[20]	IS (38) vs Con (50)	Plasma	24 h	miR-30a score	Down (2.046)	--	0.910 (0.869-0.979)	1.675	94.0	80.0	--	Relative expression	U6, $2^{-\Delta\Delta CT}$	RT-qPCR
21	Wang 2017[34]	IS (36) vs Con (25)	Plasma	3 h	lncRNA H19	Up	NIHSS (3 h post stroke), r = 0.1964, p = 0.0068; NIHSS (7 d post stroke), r = 0.6488, p < 0.0001	0.910	--	80.6	92.0	--	Relative expression	β-actin	RT-qPCR
22	Gui 2019[54]	IS (CE, 51) vs Con (33)	Plasma	24 h	miR-125b	Up	--	0.906 (0.888-0.956)	--	86.0	87.0	--	Relative expression	Spiked-in cel-miR-39, $2^{-\Delta\Delta CT}$	RT-qPCR
23	Toor 2023[31]	IS (95) vs TIA (30)	Serum	24 h	Panels of 25 miRNA (classifier model, Up (miR-3158-3p, miR-548c-5p, miR-132-3p, miR-20a-5p, miR-18a-5p, miR-484, miR-652-3p, miR-486-3p, miR-24-3p, miR-181a-5p, miR-374a-5p, miR-451a, miR-92a-3p, miR-32-5p, miR-363-	Up	--	0.901	--	--	--	--	Relative expression	RNA sequencing	RNA sequencing

					3p, miR-361-5p, miR-130a-3p, miR-222-3p, miR-3158-3p); Down (miR-664a-5p, miR-500a-3p, miR-30e-3p, miR-342-5p, miR-206, miR-184))										
24	Tiedt 2017[30]	IS (200) vs Con (100)	Plasma	24 h	miR-125a-5p, miR-125b-5p, miR-143-3p	Up	--	0.900	--	85.6	76.3	--	Relative expression	Spiked-in UniSp2, UniSp4 and UniSp5, ΔΔCq	RT-qPCR
25	Zhao 2016[44]	IS (168) vs Con (104)	Plasma	24 h	miR-335	Down	NIHSS, r = -0.3682, P < 0.001	0.898 (0.855 - 0.931)	--	97.6	69.2	0.79 (0.68 - 0.87)	Relative expression	Spiked-in cel-miR-39, $2^{-\Delta CT}$	RT-qPCR
26	Jia 2015[10]	IS (146) vs Con (96)	Serum	24 h	miR-145-5p, CRP	Up	miR-145 is correlated with NIHSS (r = 0.6288, p < 0.0001) and Infarct volume by MRI (r = 0.6249, p < 0.0001).	0.896 (0.804-0.951)	--	--	--	--	Relative expression	U6, $2^{-\Delta\Delta CT}$	RT-qPCR
27	Wang 2014[36]	IS (MRI-, 60) vs Con (116)	Plasma	24 h	miR-4306	Up	--	0.877 (0.799-0.954)	--	--	--	--	Relative expression	RNU6B, $2^{-\Delta\Delta CT}$	RT-qPCR
28	Chen 2020[61]	IS (215) vs RC (215)	Plasma	24 h	lncRNA HULC	Up	NIHSS, r = 0.456, p < 0.001	0.876	1.508	80.9	82.8	--	Relative expression	GAPDH, $2^{-\Delta\Delta CT}$	RT-qPCR

								(0.843-0.908)							
29	Nguyen 2020[22]	IS (20) vs Con (20)	Plasma	24 h	tRNA-TyrGTA, tRNA-ThrCGT and tRNA-ValCAC	--	--	0.875 (0.759-0.991)	--	80.0	90.0	--	Relative expression	RNA-sequencing	RNA-sequencing
30	Zuo 2020[51]	IS (200) vs Con (100)	Plasma	72 h	circFUNDC1, circPDS5B, circCDC14A	Up	--	0.875	--	--	--	--	Copy number/ µL	Absolute quantification analysis	RT-qPCR
31	Eyiletén 2022[6]	IS (28) vs Con (35)	Plasma	24 h	let-7f-5p	Down (0.0087)	--	0.874 (0.76-0.99)	--	--	--	--	Relative expression	Spiked-in cel-miR-39, $2^{-\Delta CT}$	RT-qPCR
32	Gui 2019[54]	IS (LAA, 26) vs Con (33)	Plasma	24 h	miR-7-2-3p	Up	--	0.874 (0.795-0.945)	--	86.0	87.0	--	Relative expression	Spiked-in cel-miR-39, $2^{-\Delta\Delta CT}$	RT-qPCR
33	Zuo 2020[51]	IS (200) vs Con (100)	Plasma	72 h	circCDC14A	Up	Infarct volume, r = 0.04591, p = 0.006	0.872	--	--	--	--	Copy number/ µL	Absolute quantification analysis	RT-qPCR
34	Wu 2019[59]	IS (71) vs RC (71)	Plasma	48 h	circTLK1	Up	Infarct volume by MRI, r = 0.7197, p = 0.0017	0.868	2.207	78.9	91.5	--	Relative expression	GAPDH	RT-qPCR
35	Ewida 2021[5]	IS (50) vs HS (25)	Serum	24 h	lncRNA HIF1A-AS2	Down	--	0.867	7.830	80.0	82.0	--	Relative expression	GAPDH, $2^{-\Delta\Delta CT}$	RT-qPCR
36	Zhou 2021[49]	IS (108) vs Con (108)	Serum	72 h	miR-124	Down	Low miR-124 expression correlated with poor survival prognosis	0.8668	--	93.5	71.3	--	Relative expression	U6, $2^{-\Delta\Delta CT}$	RT-qPCR

37	Chen 2018[3]	IS (128) vs Con (102)	Serum	24 h	miR-146b, IL-6, hs-CRP	--	--	0.866 (0.802-0.925)	--	--	--	--	Relative expression	U6, $2^{-\Delta\Delta CT}$	RT-qPCR
38	Gui 2019[54]	IS (CE, 51) vs Con (33)	Plasma	24 h	miR-125a	Up	--	0.866 (0.795-0.963)	--	87.0	82.0	--	Relative expression	Spiked-in cel-miR-39, $2^{-\Delta\Delta CT}$	RT-qPCR
39	Chen 2018[3]	IS (128) vs Con (102)	Serum	24 h	miR-146b, hs-CRP	--	--	0.863 (0.801-0.936)	--	--	--	--	Relative expression	U6, $2^{-\Delta\Delta CT}$	RT-qPCR
40	Peng 2015[24]	IS (72) vs Con (51)	Serum	24 h	let-7e	Up	--	0.86 (0.754-0.968)	0.760	82.8	73.4	--	Relative expression	18S rRNA, $2^{-\Delta\Delta CT}$	RT-qPCR
41	Chen 2017[2]	IS (50) vs Con (33)	Serum-derived exosomes	72 h	miR-223	Up (2.754)	NIHSS: r = 0.31, p = 0.03	0.859	-0.500	84.0	78.8	1.70 (1.31-2.20)	Relative expression	miR-16, CT method	RT-qPCR
42	Gui 2019[54]	IS (LAA, 26) vs Con (33)	Plasma	24 h	miR-7-2-3p	Up	--	0.849 (0.777-0.917)	--	84.0	83.0	--	Relative expression	Spiked-in cel-miR-39, $2^{-\Delta\Delta CT}$	RT-qPCR
43	Zuo 2020[51]	IS (200) vs Con (100)	Plasma	72 h	circPDS5B	Up	Infarct volume, r = 0.02594, p = 0.04	0.841	--	--	--	--	Copy number/ µL	Absolute quantification analysis	RT-qPCR
44	Song 2021[28]	IS (80) vs RC (30)	Plasma	9 h	miR-409-3p	Up	NIHSS, r = 0.865, p < 0.001	0.835	4.760	89.5	93.8	--	Relative expression	$2^{-\Delta\Delta Cq}$	RT-qPCR
45	Zhou 2018[47]	IS (50) vs Con (50)	Serum-derived exosomes	24 h	miR-134	Up	NIHSS, r = 0.6079, p = 0.000064; Infarct volume by MRI, r = 0.7841, p = 0.00043	0.834 (0.88-0.880.97)	--	75.3	72.8	--	Relative expression	$2^{-\Delta\Delta CT}$	RT-qPCR

46	Gui 2019[54]	IS (CE, 51) vs Con (33)	Plasma	24 h	let-7b	Up	--	0.833 (0.763-0.925)	--	83.0	85.0	--	Relative expression	Spiked-in cel-miR-39, $2^{-\Delta\Delta CT}$	RT-qPCR
47	Ewida 2021[5]	IS (50) vs HS (25)	Serum	24 h	mRNA HIF1-a	Down	--	0.83	7.170	80.0	84.0	--	Relative expression	GAPDH, $2^{-\Delta\Delta CT}$	RT-qPCR
48	Wang 2018[35]	IS (15) vs Con (24)	Plasma-derived exosomes	6 h	miR-30a-5p	Up (5.7)	--	0.826 (0.665-0.988)	--	--	--	--	Relative expression	U6, $2^{-\Delta\Delta CT}$	RT-qPCR
49	AdlySadik 2021[1]	IS (46) vs Con (50)	Serum	24 h	mRNA STAT3	Up (4.2)	--	0.82	2.050	83.3	67.8	--	Relative expression	β-actin, ΔCT	RT-qPCR
50	Chen 2018[3]	IS (128) vs Con (102)	Serum	24 h	miR-146b, IL-6	--	--	0.819 (0.738-0.892)	--	--	--	--	Relative expression	U6, $2^{-\Delta\Delta CT}$	RT-qPCR
51	Jia 2015[10]	IS (146) vs Con (96)	Serum	24 h	miR-221-5p, CRP	Down	--	0.819 (0.738-0.892)	--	--	--	--	Relative expression	U6, $2^{-\Delta\Delta CT}$	RT-qPCR
52	Rahmati 2020[25]	IS (52) vs RC (52)	Serum	12 h	miR-602	Down	--	0.8168 (0.7281-0.9054)	0.129	78.8	84.6	--	Relative expression	U6, $2^{-\Delta CT}$	RT-qPCR
53	Jia 2015[10]	IS (146) vs Con (96)	Serum	24 h	miR-23a-3p, CRP	Down	--	0.816 (0.726-0.887)	--	--	--	--	Relative expression	U6, $2^{-\Delta\Delta CT}$	RT-qPCR
54	Gui 2019[54]	IS (LAA, 26) vs Con (33)	Plasma	24 h	miR-1908	Down	--	0.811 (0.734-0.879)	--	79	83	--	Relative expression	Spiked-in cel-miR-39, $2^{-\Delta\Delta CT}$	RT-qPCR
55	Wang 2017[38]	IS (78) vs Con (39)	Serum	6 h	miR-221-3p	Down	--	0.8106 (0.7252-0.8960)	--	--	--	--	Relative expression	U6, $2^{-\Delta CT}$	RT-qPCR

56	Cheng 2018[4]	IS (77) vs Con (42)	Serum	24 h	miR-148b, miR-27b-3p	--	--	0.8103 (0.7006-0.9199)	--	67.2	92.9	--	Relative expression	U6, $2^{-\Delta CT}$	RT-qPCR
57	Wang 2022[37]	IS (200) vs Con (100)	Plasma	72 h	circPTP4A2, circTLK2	Up	--	0.805 (0.751-0.860)	--	90.0	63.0	--	Relative expression	GAPDH, $2^{-\Delta\Delta CT}$	RT-qPCR
58	Li 2020[15]	IS (210) vs RC (210)	Plasma	48 h	lncRNA NEAT1	Up (2.135)	NIHSS, r = 0.503, p < 0.001	0.804 (0.763-0.845)	1.471	64.3	82.9	--	Relative expression	GAPDH, $2^{-\Delta\Delta CT}$	RT-qPCR
59	Zhao 2014[46]	IS (80) vs Con (30)	Serum	48 h	miR-210	Down	--	0.804 (0.700 - 0.908)	--	90.4	76.2	--	Relative expression	$2^{-\Delta\Delta CT}$	RT-qPCR
60	Ji 2016[9]	IS (65) vs Con (66)	Serum-derived exosomes	24 h	miR-9-3p	Up (16)	NIHSS: r = 0.7126, p < 0.01; Infarct volume by MRI: r = 0.6768, p < 0.01	0.8026 (0.7235-0.8816)	--	--	--	--	Relative expression	Spiked-in cel-miR-39, Target normalized = Target raw-(Control raw-Control median run)	RT-qPCR
61	Zuo 2020[51]	IS (200) vs Con (100)	Plasma	72 h	circFUNDC1	Up	Infarct volume, r = 0.02747, p = 0.0345	0.796	--	--	--	--	Copy number/ µL	Absolute quantification analysis	RT-qPCR
62	Tian 2016[29]	IS (PACI, 17) vs RC (23)	Plasma	6 h	miR-16	Up	--	0.795 (0.642-0.948)	2.168	70.6	87.0	--	Relative expression	Spiked-in cel-miR-54, $2^{-\Delta CT}$	RT-qPCR
63	Gui 2019[54]	IS (LAA, 26) vs Con (33)	Plasma	24 h	miR-1908	Down	--	0.789 (0.715-0.867)	--	77	81	--	Relative expression	Spiked-in cel-miR-39, $2^{-\Delta\Delta CT}$	RT-qPCR

64	AdlySadik 2021[1]	IS (46) vs Con (50)	Serum	24 h	mRNA JAK2	Up (2.9)	--	0.78	1.250	61.9	84.2	--	Relative expression	β-actin, ΔCT	RT-qPCR
65	Chen 2018[3]	IS (128) vs Con (102)	Serum	24 h	miR-146b	Up	NIHSS: r = 0.646, p < 0.0001; Infarct volume by MRI: r = 0.5199, p < 0.0001	0.776 (0.628-0.813)	--	--	--	--	Relative expression	U6, $2^{-\Delta\Delta CT}$	RT-qPCR
66	Tian 2016[29]	IS (33) vs RC (23)	Plasma	6 h	miR-16	Up	--	0.775 (0.653-0.897)	1.833	69.7	87.0	--	Relative expression	Spiked-in cel-miR-54, $2^{-\Delta CT}$	RT-qPCR
67	Cheng 2018[4]	IS (77) vs Con (42)	Serum	24 h	miR-148b, miR-151b, miR-27b-3p	--	--	0.7712 (0.6520-0.8903)	--	52.4	91.7	--	Relative expression	U6, $2^{-\Delta CT}$	RT-qPCR
68	Kijpaisalratana 2020[12]	IS (23) vs SM (35)	Serum	72 h	miR-125b-5p	Up (2.572)	Infarct volume, r = 0.546, p = 0.007	0.7689 (0.6418-0.8961)	89.000	78.3	74.3	--	copies/μL	Absolute quantification analysis	RT-qPCR
69	Jin 2017[11]	IS (106) vs RC (110)	Plasma	24 h	miR-126, miR-130a, miR222, miR-218, miR-185	--	--	0.767 (0.705-0.829)	--	87.7	54.5	--	Relative expression	U6, $2^{-\Delta\Delta CT}$	RT-qPCR
70	Kijpaisalratana 2020[12]	IS (23) vs SM (35)	Serum	72 h	miR-125a-5p, miR-125b-5p	--	--	0.7665 (0.6382-0.8948)	--	--	--	--	copies/μL	Absolute quantification analysis	RT-qPCR
71					miR-125a-5p, miR-125b-5p, miR-433-5p			0.7627 (0.6345-0.8910)							

72	Wang 2022[37]	IS (200) vs Con (100)	Plasma	72 h	circPTP4A2	Up	--	0.762 (0.703-0.820)	--	69.5	82.0	2.788 (1.876 - 4.145)	Relative expression	GAPDH, $2^{-\Delta\Delta CT}$	RT-qPCR
73	Feng 2019[7]	IS (126) vs RC (125)	Plasma	24 h	lncRNA ANRIL	Down (0.36 folds)	NIHSS: r = -0.351, p < 0.001	0.759 (0.741-0.849)	--	72.2	71.2	--	Relative expression	U6, $2^{-\Delta\Delta CT}$	RT-qPCR
74	Kijpaisalratana 2020[12]	IS (23) vs SM (35)	Serum	72 h	miR-125b-5p, miR-433-5p	--	--	0.759 (0.6282-0.8899)	--	--	--	--	copies/μL	Absolute quantification analysis	RT-qPCR
75	Eyileten 2022[6]	IS (28) vs Con (35)	Plasma	24 h	miR-19a-3p	Up (6.026)	--	0.755 (0.63-0.88)	--	--	--	--	Relative expression	Spiked-in cel-miR-39, $2^{-\Delta CT}$	RT-qPCR
76	Kijpaisalratana 2020[12]	IS (23) vs SM (35)	Serum	72 h	miR-125a-5p	Up (2.326)	Infarct volume, r = 0.530, p = 0.009	0.7516 (0.6196-0.8835)	299.00	87.0	57.1	--	copies/μL	Absolute quantification analysis	RT-qPCR
77					miR-125a-5p, miR-433-5p	--	--	0.7503 (0.6171-0.8835)	--	--	--				
78	Wang 2017[38]	IS (78) vs Con (39)	Serum	6 h	miRNA-382-5p	Down	--	0.7483 (0.6300-0.8665)	--	--	--	--	Relative expression	U6, $2^{-\Delta CT}$	RT-qPCR
79	Wang 2018[35]	IS (32) vs Con (24)	Plasma-derived exosomes	> 14 days	miR-21-5p	Up (1.8)	--	0.734 (0.596-0.871)	--	--	--	--	Relative expression	U6, $2^{-\Delta\Delta CT}$	RT-qPCR
80	Wang 2022[37]	IS (200) vs Con (100)	Plasma	72 h	circTLK2	Up	--	0.734 (0.675-0.793)	--	66.5	73.0	2.480 (1.676 - 3.670)	Relative expression	GAPDH, $2^{-\Delta\Delta CT}$	RT-qPCR

81	Nguyen 2020[22]	IS (20) vs Con (20)	Plasma	24 h	tRNA-TyrGTA	--	--	0.731 (0.566-0.897)	--	--	--	--	Relative expression	RNA-sequencing	RNA-sequencing
82	Cheng 2018[4]	IS (77) vs Con (42)	Serum	24 h	miR-148b, miR-151b	--	--	0.7266 (0.5856-0.8675)	--	65.6	91.7	--	Relative expression	U6, $2^{-\Delta CT}$	RT-qPCR
83					miR-151b, miR-27b-3p			0.7143 (0.5763-0.8522)	--	27.1	93.3				RT-qPCR
84	Kijpaisalratana 2020[12]	IS (23) vs SM (35)	Serum	72 h	miR-433-5p	Up (1.724)	--	0.7143 (0.5780-0.8506)	46.000	87.0	48.6	--	copies/μL	Absolute quantification analysis	RT-qPCR
85	Wang 2018[35]	IS (31) vs Con (24)	Plasma-derived exosomes	8 - 14 days	miR-21-5p	Up (1.7)	--	0.714 (0.570-0.859)	--	--	--	--	Relative expression	U6, $2^{-\Delta\Delta CT}$	RT-qPCR
86	Wang 2022[37]	IS (200) vs TIA (66)	Plasma	72 h	circPTP4A2, circTLK2	Up	--	0.707 (0.640-0.773)	--	57.0	87.9	--	Relative expression	GAPDH, $2^{-\Delta\Delta CT}$	RT-qPCR
87					circPTP4A2	Up		0.704 (0.637-0.770)	--	56.0	87.9	1.713 (1.220 - 2.405)			
88	Nguyen 2020[22]	IS (20) vs Con (20)	Plasma	24 h	tRNA-ValCAC	--	--	0.703 (0.523-0.882)	--	--	--	--	Relative expression	RNA-sequencing	RNA-sequencing
89	Leung 2014[13]	IS (93) vs HS (19)	Plasma	24 h	miR-124-3p	Down (0.526)	Infarct volume, r = 0.809, p = 0.0005 (in HS group)	0.7 (0.59-0.79)	> 3 × 10^5	68.4	71.2	--	copies/mL	Absolute quantification analysis	RT-qPCR

90	Ji 2016[9]	IS (65) vs Con (66)	Serum-derived exosomes	24 h	miR-124-3p	Up (4 folds)	NIHSS: r = 0.6825, p < 0.01; Infarct volume by MRI: r = 0.6312, p < 0.01	0.6976 (0.6506-0.7895)	--	--	--	--	Relative expression	Spiked-in cel-miR-39, Target normalized = Target raw-(Control raw-Control median run)	RT-qPCR
91	Nguyen 2020[22]	IS (20) vs Con (20)	Plasma	24 h	tRNA-ThrCGT	--	--	0.695 (0.529-0.861)	--	--	--	--	Relative expression	RNA-sequencing	RNA-sequencing
92	Kijpaisalratana 2020[12]	IS (23) vs SM (35)	Serum	72 h	miR-143-3p	Up (1.864)	--	0.6919 (0.5523-0.8315)	--	--	--	--	copies/µL	Absolute quantification analysis	RT-qPCR
93					miR-151b	Up		0.6852 (0.5412-0.8291)	--	43.1	93.3				RT-qPCR
94	Cheng 2018[4]	IS (77) vs Con (42)	Serum	24 h	miR-27b-3p	Up	--	0.6657 (0.5306-0.8008)	--	50.0	78.9	--	Relative expression	U6, $2^{-\Delta CT}$	RT-qPCR
95					miR-148b-3p	Down		0.6647 (0.4895-0.8399)	--	51.5	80.0				RT-qPCR
96	Leung 2014[13]	IS (93) vs HS (19)	Plasma	24 h	miR-16	Up (1.231)	--	0.66 (0.55-0.76)	≤ 2 x 10^9	94.7	35.1	--	copies/mL	Absolute quantification analysis	RT-qPCR

97	Jin 2017[11]	IS (106) vs RC (110)	Plasma	24 h	miR-126	Down	NIHSS, r = -0.398, p < 0.001	0.654 (0.580-0.728)	--	--	--	0.840 (0.766 - 0.922)	Relative expression	U6, $2^{-\Delta\Delta CT}$	RT-qPCR
98	Kijpaisalratana 2020[12]	IS (23) vs SM (35)	Serum	72 h	miR-376a-3p	p > 0.05	Infarct volume, r = 0.423, p = 0.044	0.6497 (0.5042-0.7952)	--	--	--	--	copies/μL	Absolute quantification analysis	RT-qPCR
99	Jin 2017[11]	IS (106) vs RC (110)	Plasma	24 h	miR-130a	Down	--	0.642 (0.568-0.175)	--	--	--	0.885(0.827 - 0.948)	Relative expression	U6, $2^{-\Delta\Delta CT}$	RT-qPCR
100	Wang 2022[37]	IS (200) vs TIA (66)	Plasma	72 h	circTLK2	Up	--	0.632 (0.546-0.719)	--	90.5	37.9	1.429 (1.078 - 1.894)	Relative expression	GAPDH, $2^{-\Delta\Delta CT}$	RT-qPCR
101	Wang 2017[38]	IS (78) vs Con (39)	Serum	6 h	miRNA-4271	Up p > 0.05	--	0.6317 (0.5127-0.7508)	--	--	--	--	Relative expression	U6, $2^{-\Delta CT}$	RT-qPCR
102	Jin 2017[11]	IS (106) vs RC (110)	Plasma	24 h	miR-218	Up	--	0.624 (0.549-0.699)	--	--	--	1.138 (1.036 - 1.250)	Relative expression	U6, $2^{-\Delta\Delta CT}$	RT-qPCR
103	Rahmati 2021[26]	IS (52) vs Con (52)	Serum	24 h	miR-210	Down	--	0.6106	0.129	59.6	65.4	--	Relative expression	U6, $2^{-\Delta CT}$	RT-qPCR
104	Jin 2017[11]	IS (106) vs RC (110)	Plasma	24 h	miR-185	Up	--	0.601 (0.525-0.676)	--	--	--	1.099 (1.003 - 1.205)	Relative expression	U6, $2^{-\Delta\Delta CT}$	RT-qPCR
105					miR222	Up	NIHSS, r = 0.698, p < 0.001	0.584 (0.508-0.661)				1.064 (1.004 - 1.126)			RT-qPCR
106	Kijpaisalratana	IS (23) vs SM (35)	Serum	72 h	miR-342-3p	p > 0.05	--	0.5193	--	--	--	--	copies/μL	Absolute quantific	RT-qPCR

	2020[12]							(0.3674-0.6712)						ation analysis	
107	Wang 2018[35]	IS (33) vs Con (24)	Plasma-derived exosomes	72 h	miR-30a-5p	Down (0.5)	--	0.438 (0.240-0.635)	--	--	--	--	Relative expression	U6, $2^{-\Delta\Delta CT}$	RT-qPCR

Notes: Control (Con), Healthy control (HC), Risk control (RC), Stroke mimics (SM), Ischemic stroke (IS), Hemorrhagic stroke (HS), Large-artery atherosclerosis (LAA), National Institutes of Health Stroke Scale (NIHSS), Magnetic Resonance Imaging (MRI), Total Anterior Cerebral Infarction (TACI), Cardioembolism (CE), Posterior Cerebral Infarction (PACI), Transient Ischemic Attack (TIA).

Supplemental Table S4. The statistical information of cfRNA in the included studies without AUC

NO.	Study ID	Biomarkers	Groups	Specimen	Sampling time	Level (fold change)	Association	Unit	Normalization	Method
1	Ishida 2020[8]	tRNA derivatives	IS (75) vs RC (22)	Plasma	24 h	Up (2.70)	Infarct volume by MRI: r = 0.445, p = 0.00018	ng/mL	anti-1mA	ELISA
2					48 h	Up (2.82)	--			
3					7 d	Up (2.27)	--			

4					30 d	Up (1.39)	--		
5	Li 2017[14]	miR-422a	IS (27) vs Con (25)	Plasma-derived exosomes	72 h	Up	--	Relative expression	U6, $2^{-\Delta\Delta CT}$	RT-qPCR
6	Li 2015[16]	miR-106b-5p	IS (53) vs RC (50)	Serum	24 h	Up (1.74)	--	Relative expression	Spiked-in syn-cel-lin-39, $2^{-\Delta\Delta CT}$	RT-qPCR
7		miR-1246				Up (1.96)				
8		miR-32-3p				Up (1.57)				
9		miR-532-5p				Down (0.613)				
10		miR-1299	IS (22) vs RC (24)			Up (1.98)				
11		miR-1913				Down (0.629)				
12		miR-224-3p				Down (0.613)				
13		miR-3149				Up (1.96)				
14		miR-423-5p				Up (1.64)				
15		miR-451a				Up (1.82)				
16		miR-4739				Up (1.83)				
17	Liu 2019[17]	miR-128	IS (36) vs Con (25)	Plasma	72 h	Up	--	Relative expression	U6, $2^{-\Delta\Delta CT}$	RT-qPCR
18	Liu 2015[19]	miR-124	IS (31) vs Con (11)	Serum	24 h	Down	Infarct volume by MRI: $r = -0.423$, $p = 0.022$	Relative expression	Spiked-in syn-cel-lin-39, $2^{-\Delta\Delta CT}$	RT-qPCR
19	Ma 2019[21]	miR-93	IS (33) vs Con (20)	Plasma	6 h	Down	--	Relative expression	U6, $2^{-\Delta\Delta CT}$	RT-qPCR
20	Sheikhbahaei 2019[27]	miR-503	IS (DM, 15) vs Con (5)	Plasma	72 h	Up (2.99)	--	Relative expression	U6	RT-qPCR
21	Xiang 2017[39]	let-7i	IS (46) vs Con (39)	Plasma	24 h	Down (0.8)	NIHSS: $r = 0.230$, $p = 0.001$	Relative expression	Spiked-in syn-cel-lin-39, $2^{-\Delta\Delta CT}$	RT-qPCR
22	Xiao 2019[40]	lncRNA H19	IS (40) vs Con (25)	Plasma	3 h	Up	NIHSS: $r = 0.6306$, $p < 0.001$	Relative expression	β-actin, $2^{-\Delta\Delta CT}$	RT-qPCR
23	Xu 2020[41]	lnc-CALM1-7	IS (100) vs RC (100)	Serum-derived exosomes	24 h	Down (0.01)	--	Relative expression	GAPDH, $2^{-\Delta\Delta CT}$	RT-qPCR
24		lnc-CRKL-2				Up				

25		lnc-NTRK3-4				Up				
26		RPS6KA2-AS1				Down				
27	Zhang 2020[42]	miR-155	IS (40) vs RC (70)	Plasma-derived microvesicles	24 h	Up (1.7)		Relative expression	$2^{-\Delta\Delta CT}$	RT-qPCR
28	Zhang 2020[43]	miR-503	IS (20) vs RC (81)	Plasma	6 h	Up	--	nmol/L	Standard curve	RT-qPCR
29			IS (39) vs RC (81)		24 h	Up				
30	Zhao 2019[45]	miR-494	IS (76) vs Con (52)	Plasma	6 h	Up	mRS at 1 year (IS = 32), r = 0.459, p = 0.008	Relative expression	U6, $2^{-\Delta\Delta CT}$	RT-qPCR
31	Zhou 2014[48]	miR-21-5p	IS (68) vs Con (21)	Plasma	24 h	Up	NIHSS, r = -0.703, p < 0.05	Relative expression	Spiked-in cel-miR-39,2 exp (mean Ct spiked-in controls - Ct target miRNA) and log transformed	RT-qPCR
32		miR-24-3p				Up	NIHSS, r = -0.694, p < 0.05			
33	Zhou 2022[50]	miR-25	IS (60) vs Con (45)	Plasma	6 h	Up	Positive link between miR-25 and mRS at 1-year post stroke	Relative expression	U6, $2^{-\Delta\Delta CT}$	RT-qPCR
34	vanKralingen 2019[55]	miR-17-5p	IS (139) vs SM (34)	Serum-derived exosomes	48 h	Up (1.54)	--	Relative expression	Spiked-in cel-miR-39, ΔΔCT	RT-qPCR
35			IS (SVD, 37) vs SM (34)			Up (2.15)				
36		miR-20b-5p	IS (139) vs SM (34)			Up (1.66)				
37			IS (SVD, 37) vs SM (34)			Up (2.36)				
38		miR-27b-3p	IS (139) vs SM (34)			Up (1.62)				
39			IS (SVD, 37) vs SM (34)			Up (2.24)				
40		miRNA-93-5p	IS (SVD, 37) vs SM (34)			Up (2.23)				
41		circDLGAP4		Plasma	24 h	Down (0.6)	--	Relative expression	18S	RT-qPCR

42	Bai 2018[58]	miR-143	IS (26) vs Con (26)			Up (5.01)			U6	
43	Kotb 2019[60]	miR-146a	IS (44) vs Con (22)	Serum	24 h	Down	GCS, r = -0.352, p = 0.022	Relative expression	snoRD86, $2^{-\Delta\Delta CT}$	RT-qPCR
44	Yang 2018[62]	miR-195	IS (96) vs Con (45)	Plasma	72 h	Down	NIHSS, r = -0.3684, p < 0.001	Relative expression	U6	RT-qPCR
45	Guo 2022[63]	miR-185	AIS (142) vs Control (50)	Serum	24 h	Up	--	Relative expression	U6, $2^{-\Delta\Delta CT}$	RT-qPCR
46		miR-424				Up				
47	Otero-Ortega 2021[64]	miR-15a-5p	IS (CSC) vs HC	Serum	24 h	Down	--	Relative expression	Spiked-in cel-miR-39	RT-qPCR
48			IS (CSC) vs SC			Down				
49		miR-100-5p	IS (CSC) vs HC			Down				
50			IS (CSC) vs SC			Down				
51		miR-199a-3p	IS-CSC vs HC			Down				
52		miR-29-3p	IS (CSC) vs HC			Up				
53			IS (SC) vs SC			Up				
54		miR-339-5p	IS (CSC) vs HC			Down				
55			IS (CSC) vs SC			Down				
56		miR-369-5p	IS (CSC) vs HC			Down				
57		miR-424-5p	IS (CSC) vs HC			Down				
58			IS (CSC) vs SC			Down				

59	Zhou 2022[65]	miR-129-1-3p	IS (LAA, 12) vs Con (15)	Plasma	6 h	Up	--	Relative expression	Microarray	Microarray
60		miR-4312				Up				
61		miR-5196-3p				Up				
62	Chen 2021[66]	lnc-OIP5-AS1	IS (15) vs Con (15)	Plasma	24 h	Down	--	Relative expression	GAPDH, $2^{-\Delta\Delta CT}$	RT-qPCR
63		miR-186-5p				Up			U6, $2^{-\Delta\Delta CT}$	
64	Gong 2016[56]	let-7c	IS (MCI, 88) vs Con (45)	Serum	48 h	Down	--	Relative expression	5 s rRNA, $2^{-\Delta\Delta CT}$	RT-qPCR
65		let-7f				Down				
66		let-7b	IS (nonMCI, 40) vs Con (45)			Up				
67		let-7c				Up				
68		let-7d				Up				
69		let-7f				Up				

Notes: Control (Con), Healthy control (HC), Risk control (RC), Ischemic stroke (IS), Acute Ischemic stroke (AIS), Stroke mimics (SM), Cortical-Subcortical (CSC), Subcortical (SC), Large-artery atherosclerosis (LAA), National Institutes of Health Stroke Scale (NIHSS), Massive Cerebral Infarction (MCI), Diabetes mellitus (DM), Small Vessel Disease (SVD).

Supplemental Table S5. Temporal changes of cfNAs in the included studies

NO.	Study ID	Groups	Specimen	Sampling time	Biomarker	Level (fold change)	AUC (95% CI)	Cut point	Sen (%)	Spe (%)	Adjusted OR	Unit
1	Peng 2015[24]	IS (63) vs Con (51)	Serum	8 d	let-7e	Up	--	--	--	--	--	Relative expression
2				15 d		Up						
3	Wang 2018[35]	IS (15) vs Con (24)	Plasma-derived exosomes	6 h	miR-30a-5p	Up (5.7)	0.826 (0.665-0.988)	--	--	--	--	Relative expression
4				72 h		Down (0.5)	0.438 (0.240-0.635)					
5			Serum	24 h	miR-124	Down	0.953	--	93.5	91.7	--	

6	Zhou 2021[49]	IS (108) vs Con (108)		48 h		Down	0.949		93.5	91.7	--	Relative expression
7				72h		Down	0.867	--	93.5	71.3	--	
8	Ishida 2020[8]	IS (75) vs RC (22)	Plasma	48 h	tRNA derivatives	Up (2.82)	--	--	--	--	--	Relative expression
9				7 d		Up (2.27)						
10				30 d		Up (1.39)						
11				48 h		Up (2.82)						
12	Zhang 2020[43]	IS (20) vs RC (81)	Plasma	6 h	miR-503	Up	--	--	--	--	--	Relative expression
13		IS (39) vs RC (81)		24 h		Up						

Notes: Control (Con), Risk control (RC), Ischemic stroke (IS), Hemorrhagic stroke (HS).

Supplemental Table S6. The statistical information of cfRNA reported twice in the included studies

NO.	Biomarker	Study ID	Comparison group	Specimen	Sampling time	Level	Association	AUC (95% CI)	Cut point	Sen (%)	Spe (%)	Unit	Method
1	miR-106b-5p	Wang 2014[36]	IS (MRI-, 60) vs Con (116)	Plasma	24 h	Up (3.63)	--	0.999 (0.997-1.000)	--	--	--	RNU6B, $2^{-\Delta\Delta CT}$	RT-qPCR
2			IS (MRI+, 76) vs Con (116)	Plasma	24 h	Up (23.9)	--	0.962 (0.930-0.993)	--	--	--		
3		Li 2015 [16]	IS (53) vs Con (50)	Serum	24 h	Up (1.74)	--	--	--	--	--	Spiked-in syn-cel-lin-39, $2^{-\Delta\Delta CT}$	RT-qPCR
4	miR-124	Zhou 2021[49]	IS (108) vs Con (108)	Serum	24 h	Down	Low miR-124 correlated with poor survival	0.953	--	93.5	91.7	U6, $2^{-\Delta\Delta CT}$	RT-qPCR
5		Liu 2015[19]	IS (31) vs Con (11)	Serum	24 h	Down	Infarct volume by MRI: r = -0.423, p = 0.022	--	--	--	--	Spiked-in cel-miR-39, $2^{-\Delta CT}$	RT-qPCR
6	miR-155	AdlySadik 2021[1]	IS (46) vs Con (50)	Serum	24 h	Up (8.5)	--	0.940	1.75	85.7	100.0	U6, ΔCT	RT-qPCR
7		Zhang 2020[42]	IS (40) vs RC (70)	Plasma-derived microvesicles	24 h	Up (1.7)	--	--	--	--	--	Relative expression	RT-qPCR
8	miR-16	Tian 2016[29]	IS (TACI, 4) vs Con (23)	Plasma	6 h	Up	--	0.978 (0.925-1.031)	--	100.0	91.3	Spiked-in cel-miR-54, $2^{-\Delta CT}$	RT-qPCR
9			IS (LAA, 9) vs Con (23)	Plasma	6 h	Up	--	0.952 (0.879-1.024)	--	100.0	91.3		RT-qPCR
10			IS (PACI, 17) vs RC (23)	Plasma	6 h	Up	--	0.795 (0.642-0.948)	2.168	70.6	87.0		RT-qPCR

11			IS (33) vs Con (23)	Plasma	6 h	Up	--	0.775 (0.653-0.897)	1.8333	69.7	87.0		RT-qPCR
12		Leung 2014[13]	IS (93) vs HS (19)	Plasma	24 h	Up (1.231)	--	0.660 (0.55-0.76)	≤ 2 x 10^9	94.7	35.1	copies/mL	RT-qPCR
13	miR-125a-5p, miR-125b-5p, miR-143-3p	Tiedt 2017[30]	IS (200) vs Con (100)	Plasma	24 h	Up	--	0.900	--	85.6	76.3	Spiked-in UniSp2, UniSp4 and UniSp5, ΔΔCq	RT-qPCR
14	miR-125a-5p	Kijpaisalratana 2020[12]	IS (23) vs SM (35)	Serum	72 h	Up (2.326)	Infarct volume, r = 0.530, p = 0.009	0.752 (0.620-0.884)	299	87.0	57.1	copies/µL	RT-qPCR
15	miR-125b-5p		IS (23) vs SM (35)	Serum	72 h	Up (2.572)	Infarct volume, r = 0.546, p = 0.007	0.769 (0.642-0.896)	89	78.3	74.3		RT-qPCR
16	miR-143-3p		IS (23) vs SM (35)	Serum	72 h	Up (1.864)	--	0.692 (0.552-0.832)	--	--	--		RT-qPCR
17	lncRNA H19	Wang 2017[34]	IS (36) vs Con (25)	Plasma	3 h	Up	NIHSS (3 h), r = 0.1964, p = 0.0068; NIHSS (7 d), r = 0.6488, p < 0.0001	0.91	--	80.6	92.0	β-actin	RT-qPCR
18		Xiao 2019[40]	IS (40) vs HC (25)	Plasma	3 h	Up	NIHSS: r = 0.6306, p < 0.001	--	--	--	--	β-actin, $2^{-\Delta\Delta CT}$	RT-qPCR
19	let-7b	Gui 2019[54]	IS (CE, 51) vs Con (33)	Plasma	24 h	Up	--	0.833 (0.763-0.925)	--	83.0	85.0	Spiked-in cel-miR-39, $2^{-\Delta\Delta CT}$	RT-qPCR
20		Gong 2016[56]	IS (nonMCI, 40) vs Con (45)	Serum	48 h	Up	--	--	--	--	--	5 s rRNA, $2^{-\Delta\Delta CT}$	RT-qPCR
21	let-7e	Gui 2019[54]	IS (CE, 51) vs Con (33)	Plasma	24 h	Up	--	0.923 (0.859-0.998)	--	89.0	90.0	Spiked-in cel-miR-39, $2^{-\Delta\Delta CT}$	RT-qPCR
22		Peng 2015[24]	IS (72) vs Con (51)	Serum	24 h	Up	--	0.86 (0.754-0.968)	0.760	82.8	73.4	18S rRNA, $2^{-\Delta\Delta CT}$	RT-qPCR

23	miR-124-3p	Leung 2014[13]	IS (93) vs HS (19)	Plasma	24 h	Down (0.526)	Infarct volume, r = 0.809, p = 0.0005 (in HS group)	0.7 (0.59-0.79)	> 3 × 10^5	68.4	71.2	copies/mL	RT-qPCR
24		Ji 2016[9]	IS (65) vs Con (66)	Serum-derived exosomes	24 h	Up (4 folds)	NIHSS: r = 0.6825, p < 0.01; Infarct volume by MRI: r = 0.6312, p < 0.01	0.6976 (0.6506-0.7895)	--	--	--	Spiked-in cel-miR-39, Target normalized = Target raw-(Control raw-Control median run)	RT-qPCR
25	miR-27b-3p	Cheng 2018[4]	IS (77) vs Con (42)	Serum	24 h	Up	--	0.6657 (0.5306-0.8008)	--	50.0	78.9	U6, $2^{-\Delta CT}$	RT-qPCR
26		Zhou 2014[48]	IS (139) vs SM (34)	Serum-derived exosomes	48 h	Up (1.62)	--	--	--	--	--	Spiked-in cel-miR-39, ΔΔCT	RT-qPCR
27	miR-503	Sheikhbahaei 2019[27]	IS (DM, 15) vs Con (5)	Plasma	72 h	Up (2.99)	--	--	--	--	--	U6	RT-qPCR
28		Zhang 2020[43]	IS (39) vs RC (81)	Plasma	24 h	Up	--	--	--	--	--	nmol/L	RT-qPCR
29	miR-185	Guo 2022[63]	AIS (142) vs Con (50)	Serum	24 h	Up	--	--	--	--	--	U6, $2^{-\Delta\Delta CT}$	RT-qPCR
30		Jin 2017[11]	IS (106) vs RC (110)	Plasma	24 h	Up	--	0.601 (0.525-0.676)	--	--	--	U6, $2^{-\Delta\Delta CT}$	RT-qPCR
31	miR-210	Zhao 2014[46]	IS (80) vs Con (30)	Serum	48 h	Down	--	0.804 (0.700 -0.908)	--	90.4	76.2	$2^{-\Delta\Delta CT}$	RT-qPCR
32		Rahmati 2021[26]	IS (52) vs Con (52)	Serum	24 h	Down	--	0.6106	0.129	59.6	65.4	U6, $2^{-\Delta CT}$	RT-qPCR

Notes: Control (Con), Healthy control (HC), Risk control (RC), Stroke mimics (SM), Ischemic stroke (IS), Hemorrhagic stroke (HS), Large-artery atherosclerosis (LAA), National Institutes of Health Stroke Scale (NIHSS), Magnetic Resonance Imaging (MRI), Total Anterior Circulation Infarct (TACI), Cardioembolic (CE), Partial Anterior Circulation Infarct (PACI), Diabetes Mellitus (DM), Massive cerebral infarction (MCI).

References

1. Adly Sadik N, Ahmed Rashed L, Ahmed Abd-El Mawla M: **Circulating miR-155 and JAK2/STAT3 Axis in Acute Ischemic Stroke Patients and Its Relation to Post-Ischemic Inflammation and Associated Ischemic Stroke Risk Factors**. *Int J Gen Med* 2021, **14**:1469-1484.
2. Chen Y, Song Y, Huang J, Qu M, Zhang Y, Geng J, Zhang Z, Liu J, Yang GY: **Increased Circulating Exosomal miRNA-223 Is Associated with Acute Ischemic Stroke**. *Front Neurol* 2017, **8**:57.
3. Chen Z, Wang K, Huang J, Zheng G, Lv Y, Luo N, Liang M, Huang L: **Upregulated Serum MiR-146b Serves as a Biomarker for Acute Ischemic Stroke**. *Cell Physiol Biochem* 2018, **45**(1):397-405.
4. Cheng X, Kan P, Ma Z, Wang Y, Song W, Huang C, Zhang B: **Exploring the potential value of miR-148b-3p, miR-151b and miR-27b-3p as biomarkers in acute ischemic stroke**. *Biosci Rep* 2018, **38**(6).
5. Ewida HA, Zayed RK, Darwish HA, Shaheen AA: **Circulating lncRNAs HIF1A-AS2 and LINLK-A: Role and Relation to Hypoxia-Inducible Factor-1α in Cerebral Stroke Patients**. *Mol Neurobiol* 2021, **58**(9):4564-4574.
6. Eyileten C, Jakubik D, Shahzadi A, Gasecka A, van der Pol E, De Rosa S, Siwik D, Gajewska M, Mirowska-Guzel D, Kurkowska-Jastrzebska I *et al*: **Diagnostic Performance of Circulating miRNAs and Extracellular Vesicles in Acute Ischemic Stroke**. *Int J Mol Sci* 2022, **23**(9).
7. Feng L, Guo J, Ai F: **Circulating long noncoding RNA ANRIL downregulation correlates with increased risk, higher disease severity and elevated pro-inflammatory cytokines in patients with acute ischemic stroke**. *J Clin Lab Anal* 2019, **33**(1):e22629.
8. Ishida T, Inoue T, Niizuma K, Konno N, Suzuki C, Inoue T, Ezura M, Uenohara H, Abe T, Tominaga T: **Prediction of Functional Outcome in Patients with Acute Stroke by Measuring tRNA Derivatives**. *Cerebrovasc Dis* 2020, **49**(6):639-646.
9. Ji Q, Ji Y, Peng J, Zhou X, Chen X, Zhao H, Xu T, Chen L, Xu Y: **Increased Brain-Specific MiR-9 and MiR-124 in the Serum Exosomes of Acute Ischemic Stroke Patients**. *PLoS One* 2016, **11**(9):e0163645.
10. Jia L, Hao F, Wang W, Qu Y: **Circulating miR-145 is associated with plasma high-sensitivity C-reactive protein in acute ischemic stroke patients**. *Cell Biochem Funct* 2015, **33**(5):314-319.

11. Jin F, Xing J: **Circulating pro-angiogenic and anti-angiogenic microRNA expressions in patients with acute ischemic stroke and their association with disease severity**. *Neurol Sci* 2017, **38**(11):2015-2023.
12. Kijpaisalratana N, Nimsamer P, Khamwut A, Payungporn S, Pisitkun T, Chutinet A, Utoomprurkporn N, Kerr SJ, Vongvasinkul P, Suwanwela NC: **Serum miRNA125a-5p, miR-125b-5p, and miR-433-5p as biomarkers to differentiate between posterior circulation stroke and peripheral vertigo**. *BMC Neurol* 2020, **20**(1):372.
13. Leung LY, Chan CP, Leung YK, Jiang HL, Abrigo JM, Wang de F, Chung JS, Rainer TH, Graham CA: **Comparison of miR-124-3p and miR-16 for early diagnosis of hemorrhagic and ischemic stroke**. *Clin Chim Acta* 2014, **433**:139-144.
14. Li DB, Liu JL, Wang W, Li RY, Yu DJ, Lan XY, Li JP: **Plasma Exosomal miR-422a and miR-125b-2-3p Serve as Biomarkers for Ischemic Stroke**. *Curr Neurovasc Res* 2017, **14**(4):330-337.
15. Li P, Duan S, Fu A: **Long noncoding RNA NEAT1 correlates with higher disease risk, worse disease condition, decreased miR-124 and miR-125a and predicts poor recurrence-free survival of acute ischemic stroke**. *J Clin Lab Anal* 2020, **34**(2):e23056.
16. Li P, Teng F, Gao F, Zhang M, Wu J, Zhang C: **Identification of circulating microRNAs as potential biomarkers for detecting acute ischemic stroke**. *Cell Mol Neurobiol* 2015, **35**(3):433-447.
17. Liu P, Han Z, Ma Q, Liu T, Wang R, Tao Z, Li G, Li F, Zhang S, Li L *et al*: **Upregulation of MicroRNA-128 in the Peripheral Blood of Acute Ischemic Stroke Patients is Correlated with Stroke Severity Partially through Inhibition of Neuronal Cell Cycle Reentry**. *Cell Transplant* 2019, **28**(7):839-850.
18. Liu Y, Li Y, Zang J, Zhang T, Li Y, Tan Z, Ma D, Zhang T, Wang S, Zhang Y *et al*: **CircOGDH Is a Penumbra Biomarker and Therapeutic Target in Acute Ischemic Stroke**. *Circ Res* 2022, **130**(6):907-924.
19. Liu Y, Zhang J, Han R, Liu H, Sun D, Liu X: **Downregulation of serum brain specific microRNA is associated with inflammation and infarct volume in acute ischemic stroke**. *J Clin Neurosci* 2015, **22**(2):291-295.
20. Long G, Wang F, Li H, Yin Z, Sandip C, Lou Y, Wang Y, Chen C, Wang DW: **Circulating miR-30a, miR-126 and let-7b as biomarker for ischemic stroke in humans**. *BMC Neurol* 2013, **13**:178.
21. Ma Q, Li G, Tao Z, Wang J, Wang R, Liu P, Luo Y, Zhao H: **Blood microRNA-93 as an indicator for diagnosis and prediction of functional recovery of acute stroke patients**. *J Clin Neurosci* 2019, **62**:121-127.
22. Nguyen TTM, van der Bent ML, Wermer MJH, van den Wijngaard IR, van Zwet EW, de Groot B, Quax PHA, Kruyt ND, Nossent AY: **Circulating tRNA Fragments as a Novel Biomarker Class to Distinguish Acute Stroke Subtypes**. *Int J Mol Sci* 2020, **22**(1).
23. O'Connell GC, Petrone AB, Tennant CS, Lucke-Wold N, Kabbani Y, Tarabishy AR, Chantler PD, Barr TL: **Circulating extracellular DNA levels are acutely elevated in ischaemic stroke and associated with innate immune system activation**. *Brain Inj* 2017, **31**(10):1369-1375.
24. Peng G, Yuan Y, Wu S, He F, Hu Y, Luo B: **MicroRNA let-7e Is a Potential Circulating Biomarker of Acute Stage Ischemic Stroke**. *Transl Stroke Res* 2015, **6**(6):437-445.
25. Rahmati M, Azarpazhooh MR, Ehteram H, Ferns GA, Ghayour-Mobarhan M, Ghannadan H, Mobarra N: **The elevation of S100B and downregulation of circulating miR-602 in the sera of ischemic stroke (IS) patients: the emergence of novel diagnostic and prognostic markers**. *Neurol Sci* 2020, **41**(8):2185-2192.

26. Rahmati M, Ferns GA, Mobarra N: **The lower expression of circulating miR-210 and elevated serum levels of HIF-1α in ischemic stroke; Possible markers for diagnosis and disease prediction**. *J Clin Lab Anal* 2021, **35**(12):e24073.
27. Sheikhbahaei S, Manizheh D, Mohammad S, Hasan TM, Saman N, Laleh R, Mahsa M, Sanaz AK, Shaghayegh HJ: **Can MiR-503 be used as a marker in diabetic patients with ischemic stroke?** *BMC Endocr Disord* 2019, **19**(1):42.
28. Song XD, Li SX, Zhu M: **Plasma miR-409-3p promotes acute cerebral infarction via suppressing CTRP3**. *Kaohsiung J Med Sci* 2021, **37**(4):324-333.
29. Tian C, Li Z, Yang Z, Huang Q, Liu J, Hong B: **Plasma MicroRNA-16 Is a Biomarker for Diagnosis, Stratification, and Prognosis of Hyperacute Cerebral Infarction**. *PLoS One* 2016, **11**(11):e0166688.
30. Tiedt S, Prestel M, Malik R, Schieferdecker N, Duering M, Kautzky V, Stoycheva I, Böck J, Northoff BH, Klein M *et al*: **RNA-Seq Identifies Circulating miR-125a-5p, miR-125b-5p, and miR-143-3p as Potential Biomarkers for Acute Ischemic Stroke**. *Circ Res* 2017, **121**(8):970-980.
31. Toor SM, Aldous EK, Parray A, Akhtar N, Al-Sarraj Y, Abdelalim EM, Arredouani A, El-Agnaf O, Thornalley PJ, Pananchikkal SV *et al*: **Circulating MicroRNA Profiling Identifies Distinct MicroRNA Signatures in Acute Ischemic Stroke and Transient Ischemic Attack Patients**. *Int J Mol Sci* 2022, **24**(1).
32. Vallés J, Lago A, Santos MT, Latorre AM, Tembl JI, Salom JB, Nieves C, Moscardó A: **Neutrophil extracellular traps are increased in patients with acute ischemic stroke: prognostic significance**. *Thromb Haemost* 2017, **117**(10):1919-1929.
33. Vasilyeva I, Bespalov V, Baranova A, Voznyuk I, Baranenko D: **Differential Dynamics of the Levels of Low Molecular Weight DNA Fragments in the Plasma of Patients With Ischemic and Hemorrhagic Strokes**. *Basic Clin Neurosci* 2020, **11**(6):805-810.
34. Wang J, Zhao H, Fan Z, Li G, Ma Q, Tao Z, Wang R, Feng J, Luo Y: **Long Noncoding RNA H19 Promotes Neuroinflammation in Ischemic Stroke by Driving Histone Deacetylase 1-Dependent M1 Microglial Polarization**. *Stroke* 2017, **48**(8):2211-2221.
35. Wang W, Li DB, Li RY, Zhou X, Yu DJ, Lan XY, Li JP, Liu JL: **Diagnosis of Hyperacute and Acute Ischaemic Stroke: The Potential Utility of Exosomal MicroRNA-21-5p and MicroRNA-30a-5p**. *Cerebrovasc Dis* 2018, **45**(5-6):204-212.
36. Wang W, Sun G, Zhang L, Shi L, Zeng Y: **Circulating microRNAs as novel potential biomarkers for early diagnosis of acute stroke in humans**. *J Stroke Cerebrovasc Dis* 2014, **23**(10):2607-2613.
37. Wang XZ, Li S, Liu Y, Cui GY, Yan FL: **Construction of circRNA-Mediated Immune-Related ceRNA Network and Identification of Circulating circRNAs as Diagnostic Biomarkers in Acute Ischemic Stroke**. *J Inflamm Res* 2022, **15**:4087-4104.
38. Wang Y, Ma Z, Kan P, Zhang B: **The Diagnostic Value of Serum miRNA-221-3p, miRNA-382-5p, and miRNA-4271 in Ischemic Stroke**. *J Stroke Cerebrovasc Dis* 2017, **26**(5):1055-1060.
39. Xiang W, Tian C, Lin J, Wu X, Pang G, Zhou L, Pan S, Deng Z: **Plasma let-7i and miR-15a expression are associated with the effect of recombinant tissue plasminogen activator treatment in acute ischemic stroke patients**. *Thromb Res* 2017, **158**:121-125.
40. Xiao Z, Qiu Y, Lin Y, Medina R, Zhuang S, Rosenblum JS, Cui J, Li Z, Zhang X, Guo L: **Blocking lncRNA H19-miR-19a-Id2 axis attenuates hypoxia/ischemia induced neuronal injury**. *Aging (Albany NY)* 2019, **11**(11):3585-3600.
41. Xu X, Zhuang C, Chen L: **Exosomal Long Non-Coding RNA Expression from Serum of Patients with Acute Minor Stroke**. *Neuropsychiatr Dis Treat* 2020, **16**:153-160.

42. Zhang H, Chen G, Qiu W, Pan Q, Chen Y, Chen Y, Ma X: **Plasma endothelial microvesicles and their carrying miRNA-155 serve as biomarkers for ischemic stroke**. *J Neurosci Res* 2020, **98**(11):2290-2301.
43. Zhang H, Pan Q, Xie Z, Chen Y, Wang J, Bihl J, Zhong W, Chen Y, Zhao B, Ma X: **Implication of MicroRNA503 in Brain Endothelial Cell Function and Ischemic Stroke**. *Transl Stroke Res* 2020, **11**(5):1148-1164.
44. Zhao B, Zhu Z, Hao J, Wan Z, Guo X: **Decreased plasma miR-335 expression in patients with acute ischemic stroke and its association with calmodulin expression**. *J Int Med Res* 2016, **44**(6):1331-1338.
45. Zhao H, Li G, Zhang S, Li F, Wang R, Tao Z, Ma Q, Han Z, Yan F, Fan J *et al*: **Inhibition of histone deacetylase 3 by MiR-494 alleviates neuronal loss and improves neurological recovery in experimental stroke**. *J Cereb Blood Flow Metab* 2019, **39**(12):2392-2405.
46. Zhao J, Gao B, Zhai B: **[Expression and its significance of microRNA-210 in serum in acute cerebral infarction]**. *Zhonghua Wei Zhong Bing Ji Jiu Yi Xue* 2014, **26**(12):910-913.
47. Zhou J, Chen L, Chen B, Huang S, Zeng C, Wu H, Chen C, Long F: **Increased serum exosomal miR-134 expression in the acute ischemic stroke patients**. *BMC Neurol* 2018, **18**(1):198.
48. Zhou J, Zhang J: **Identification of miRNA-21 and miRNA-24 in plasma as potential early stage markers of acute cerebral infarction**. *Mol Med Rep* 2014, **10**(2):971-976.
49. Zhou X, Qi L: **miR-124 Is Downregulated in Serum of Acute Cerebral Infarct Patients and Shows Diagnostic and Prognostic Value**. *Clin Appl Thromb Hemost* 2021, **27**:10760296211035446.
50. Zhou X, Qiao B: **Inhibition of HDAC3 and ATXN3 by miR-25 prevents neuronal loss and ameliorates neurological recovery in cerebral stroke experimental rats**. *J Physiol Biochem* 2022, **78**(1):139-149.
51. Zuo L, Zhang L, Zu J, Wang Z, Han B, Chen B, Cheng M, Ju M, Li M, Shu G *et al*: **Circulating Circular RNAs as Biomarkers for the Diagnosis and Prediction of Outcomes in Acute Ischemic Stroke**. *Stroke* 2020, **51**(1):319-323.
52. Rainer TH, Wong KS, Lam W, Lam NY, Graham CA, Lo YM: **Comparison of plasma beta-globin DNA and S-100 protein concentrations in acute stroke**. *Clin Chim Acta* 2007, **376**(1-2):190-196.
53. Bustamante A, Mancha F, Macher HC, García-Berrocoso T, Giralt D, Ribó M, Guerrero JM, Montaner J: **Circulating cell-free DNA is a predictor of short-term neurological outcome in stroke patients treated with intravenous thrombolysis**. *J Circ Biomark* 2016, **5**:1849454416668791.
54. Gui Y, Xu Z, Jin T, Zhang L, Chen L, Hong B, Xie F, Lv W, Hu X: **Using Extracellular Circulating microRNAs to Classify the Etiological Subtypes of Ischemic Stroke**. *Transl Stroke Res* 2019, **10**(4):352-361.
55. van Kralingen JC, McFall A, Ord ENJ, Coyle TF, Bissett M, McClure JD, McCabe C, Macrae IM, Dawson J, Work LM: **Altered Extracellular Vesicle MicroRNA Expression in Ischemic Stroke and Small Vessel Disease**. *Transl Stroke Res* 2019, **10**(5):495-508.
56. Gong Z, Zhao S, Zhang J, Xu X, Guan W, Jing L, Liu P, Lu J, Teng J, Peng T *et al*: **Initial research on the relationship between let-7 family members in the serum and massive cerebral infarction**. *J Neurol Sci* 2016, **361**:150-157.
57. Tsai NW, Lin TK, Chen SD, Chang WN, Wang HC, Yang TM, Lin YJ, Jan CR, Huang CR, Liou CW *et al*: **The value of serial plasma nuclear and mitochondrial DNA levels in patients with acute ischemic stroke**. *Clin Chim Acta* 2011, **412**(5-6):476-479.

58. Bai Y, Zhang Y, Han B, Yang L, Chen X, Huang R, Wu F, Chao J, Liu P, Hu G *et al*: **Circular RNA DLGAP4 Ameliorates Ischemic Stroke Outcomes by Targeting miR-143 to Regulate Endothelial-Mesenchymal Transition Associated with Blood-Brain Barrier Integrity**. *J Neurosci* 2018, **38**(1):32-50.
59. Wu F, Han B, Wu S, Yang L, Leng S, Li M, Liao J, Wang G, Ye Q, Zhang Y *et al*: **Circular RNA TLK1 Aggravates Neuronal Injury and Neurological Deficits after Ischemic Stroke via miR-335-3p/TIPARP**. *J Neurosci* 2019, **39**(37):7369-7393.
60. Kotb HG, Ibrahim AH, Mohamed EF, Ali OM, Hassanein N, Badawy D, Abdelatty Aly E: **The expression of microRNA 146a in patients with ischemic stroke: an observational study**. *Int J Gen Med* 2019, **12**:273-278.
61. Chen X, Zhang X, Su C, Huang S: **Long noncoding RNA HULC in acute ischemic stroke: Association with disease risk, severity, and recurrence-free survival and relation with IL-6, ICAM1, miR-9, and miR-195**. *J Clin Lab Anal* 2020, **34**(11):e23500.
62. Yang G, Liu Z, Wang L, Chen X, Wang X, Dong Q, Zhang D, Yang Z, Zhou Q, Sun J *et al*: **MicroRNA-195 protection against focal cerebral ischemia by targeting CX3CR1**. *J Neurosurg* 2019, **131**(5):1445-1454.
63. Guo C, Yao Y, Li Q, Gao Y, Cao H: **Expression and Clinical Value of miR-185 and miR-424 in Patients with Acute Ischemic Stroke**. *Int J Gen Med* 2022, **15**:71-78.
64. Otero-Ortega L, Alonso-López E, Pérez-Mato M, Laso-García F, Gómez-de Frutos MC, Diekhorst L, García-Bermejo ML, Conde-Moreno E, Fuentes B, de Leciñana MA *et al*: **Circulating Extracellular Vesicle Proteins and MicroRNA Profiles in Subcortical and Cortical-Subcortical Ischaemic Stroke**. *Biomedicines* 2021, **9**(7).
65. Zhou B, Li B, Feng P, Wang X, Gao H, Xu L, Wang T, Guo X: **Identification of a miRNA biomarker for the large artery atherosclerosis subtype of acute ischemic stroke**. *Folia Neuropathol* 2022, **60**(2):210-220.
66. Chen Y, Liu W, Chen M, Sun Q, Chen H, Li Y: **Up-regulating lncRNA OIP5-AS1 protects neuron injury against cerebral hypoxia-ischemia induced inflammation and oxidative stress in microglia/macrophage through activating CTRP3 via sponging miR-186-5p**. *Int Immunopharmacol* 2021, **92**:107339.
